# Short timescale abnormalities in the states of spontaneous synchrony in the functional neural networks in Alzheimer's disease

**DOI:** 10.1016/j.nicl.2018.05.028

**Published:** 2018-05-22

**Authors:** Tatiana A. Sitnikova, Jeremy W. Hughes, Seppo P. Ahlfors, Mark W. Woolrich, David H. Salat

**Affiliations:** a Martinos Center for Biomedical Imaging, Massachusetts General Hospital, Charlestown, MA 02129, USA; b Harvard Medical School, Boston, MA 02115, USA; c Oxford Center for Human Brain Activity, University of Oxford, Oxford OX3 7JX, UK

**Keywords:** Alzheimer's disease, Electrophysiology, Dynamic functional connectivity, Mathematical modeling, MEG

## Abstract

Alzheimer's disease (AD) is a prevalent neurodegenerative condition that can lead to severe cognitive and functional deterioration. Functional magnetic resonance imaging (fMRI) revealed abnormalities in AD in intrinsic synchronization between spatially separate regions in the so-called default mode network (DMN) of the brain. To understand the relationship between this disruption in large-scale synchrony and the cognitive impairment in AD, it is critical to determine whether and how the deficit in the low frequency hemodynamic fluctuations recorded by fMRI translates to much faster timescales of memory and other cognitive processes. The present study employed magnetoencephalography (MEG) and a Hidden Markov Model (HMM) approach to estimate spontaneous synchrony variations in the functional neural networks with high temporal resolution. In a group of cognitively healthy (CH) older adults, we found transient (mean duration of 150–250 ms) network activity states, which were visited in a rapid succession, and were characterized by spatially coordinated changes in the amplitude of source-localized electrophysiological oscillations. The inferred states were similar to those previously observed in younger participants using MEG, and the estimated cortical source distributions of the state-specific activity resembled the classic functional neural networks, such as the DMN. In patients with AD, inferred network states were different from those of the CH group in short-scale timing and oscillatory features. The state of increased oscillatory amplitudes in the regions overlapping the DMN was visited less often in AD and for shorter periods of time, suggesting that spontaneous synchronization in this network was less likely and less stable in the patients. During the visits to this state, in some DMN nodes, the amplitude change in the higher-frequency (8–30 Hz) oscillations was less robust in the AD than CH group. These findings highlight relevance of studying short-scale temporal evolution of spontaneous activity in functional neural networks to understanding the AD pathophysiology. Capacity of flexible intrinsic synchronization in the DMN may be crucial for memory and other higher cognitive functions. Our analysis yielded metrics that quantify distinct features of the neural synchrony disorder in AD and may offer sensitive indicators of the neural network health for future investigations.

## Highlights

• Resting MEG to estimate cortical electrophysiological oscillations in older adults.
• Hidden Markov model (HMM) applied to time-courses of oscillatory amplitudes.
• HMM inferred sequential transitions between ten underlying network synchrony states.
• Visits to HMM state short-lived but resembled large-scale networks described by fMRI.
• In Alzheimer's, less frequent, less robust synchronizations in default mode network.

## 1 Introduction

Alzheimer's disease (AD) is a neurodegenerative disorder leading to severe cognitive and functional deterioration. Delineating the neural basis of the clinical symptoms in AD may help to advance its treatment. One well-established neural deficit common in AD, but not other dementias, is a disruption of self-organized synchrony between spatially separate regions in the so-called default mode network (DMN) of the brain (Agosta et al., 2012; Binnewijzend et al., 2012; Galvin et al., 2011; Gour et al., 2014; Greicius et al., 2004; Lehmann et al., 2013; Zhou et al., 2010). Present long before the onset of clinical symptoms in individuals at the genetic risk for developing AD (Machulda et al., 2011; Sheline et al., 2010a) or harboring amyloid pathology (Brier et al., 2014; Drzezga et al., 2011; Hedden et al., 2009; Sheline et al., 2010b; Wang et al., 2013), this deficit worsens as the symptoms occur and progress (Bai et al., 2008; Binnewijzend et al., 2012; Brier et al., 2012; Damoiseaux et al., 2012; Petrella et al., 2011; Westlye et al., 2011; Zhang et al., 2010), and therefore, may play a role in the etiology of the disease. To date, this DMN dysfunction in AD has been studied primarily by recording the blood oxygenation level dependent (BOLD) signal while individuals undergo task-free functional magnetic resonance imaging (fMRI). Such ‘resting’ fMRI recordings revealed an abnormally low correlation between activity time-courses of DMN regions in AD (Greicius et al., 2004). A limitation of this approach is in the sluggishness of the BOLD signal that relies on the neurovascular coupling for inferences of the brain activity (Buxton, 2013). The DMN nodes, including the inferior parietal lobule, the anterior-lateral temporal cortex, the precuneus/posterior cingulate cortex (PCC), and the medial frontal cortex, have been linked to memory and other higher cognitive functions (Addis et al., 2007; Andrews-Hanna et al., 2010; Burianova and Grady, 2007; Fox et al., 2016; Mason et al., 2007; Spreng et al., 2009; Sun et al., 2016). To determine how the disruption in large-scale synchrony between these regions may relate to the cognitive impairment in AD, it is critical to understand how deficits in the low frequency hemodynamic fluctuations recorded by fMRI translate to much faster timescales of cognition. The present study focused on the rapid network-specific fluctuations of oscillatory activity in the 4–30 Hz frequency range by using magnetoencephalography (MEG), which measures electrophysiological brain activity and offers a millisecond temporal resolution (Proudfoot et al., 2014).

The anatomic connectivity of the cerebral cortex enables short-range and long-range communication between cortical neurons, and through regenerative feedback, can support spontaneous functional organization of synchronized neuronal firing at different timescales (Buzsaki, 2006; McCormick et al., 2003; Singer, 2013). At the neuronal population level, this spontaneously orchestrated activity generates oscillations of different frequencies, which can be measured noninvasively using MEG or electroencephalography (EEG) (Buzsaki et al., 2012). Resting recordings in healthy humans have revealed that the cortical maps of inter-regional correlations, computed based on amplitude time-courses of the neural oscillations (estimated by source-localized MEG/EEG in the 2–150 Hz frequency range) or BOLD signal fluctuations (assessed by fMRI), show an intriguing spatial correspondence between the signal modalities (Brookes et al., 2011; de Pasquale et al., 2010, de Pasquale et al., 2012; Deligianni et al., 2014; Hipp and Siegel, 2015; Mantini et al., 2007). Additionally, in resting scan studies, a temporal concordance has been observed between transient EEG states of quasi-stable scalp topographies (known as ‘microstates’ and presumed to mark peaks in oscillatory [1–40 Hz] amplitudes in non-identical networks of neural sources) and the BOLD signal changes in the classic functional networks detected by simultaneously acquired fMRI (Britz et al., 2010; Jann et al., 2010; Musso et al., 2010; Yuan et al., 2012). These evident parallels suggest that, albeit the imperfect understanding of the precise relationships between the fMRI and MEG/EEG recordings, the latter might offer a promising complementary tool to study the neurophysiological basis of the DMN synchrony dysfunction in AD.

A novel analytic approach based on a Hidden Markov Model (HMM) affords a high temporal resolution method for discerning spatiotemporal patterns in fluctuating amplitudes of the electrophysiological oscillations. The Markov Model has been shown to adequately describe sequential transitions between EEG microstates (Wackermann et al., 1993). Furthermore, a recent study applied this methodology to the MEG-based estimates of cortical oscillations (4–30 Hz) during a resting scan in healthy participants to demonstrate that the HMM can independently infer a series of discrete states of neural synchrony with spatial topographies similar to the known large-scale functional networks (Baker et al., 2014). The derived states were characterized by coordinated changes in the amplitude of neural oscillations in brain regions overlapping the DMN, the dorsal attention network (DAN), the visual network (VisN), the sensorimotor network (SMN), and several other large-scale networks. Remarkably, these states tended to be very short-lived (100–200 ms long), coming in rapid succession and frequently recurring over time. However, the state time-courses also exhibited longer timescale structure, with the occurrence rates of different states varying on the timescale of several seconds. These slow rate fluctuations were comparable in frequency (<0.1 Hz) to the ultra-slow electrophysiological potentials that might reflect endogenous fluctuations of neural excitability within the functional networks (He et al., 2008; Pan et al., 2013). Previously, the ultra-slow electrophysiological potentials, as well as transient network activations, which appeared phase-locked to the ultra-slow waves, have been linked to spontaneous fMRI BOLD signal fluctuations (He et al., 2008; Matsui et al., 2016; Pan et al., 2013). It is an intriguing possibility that the rates of the rapid electrophysiological network states are influenced by the levels of cortical excitability, which could elucidate how and why the sequences of such fast events might correspond to slow BOLD signal fluctuations.

In the present study, we built on this prior successful HMM application in the MEG analysis to examine patterns of rapid intrinsic synchronizations in the large-scale neural networks in AD. We recorded MEG data in patients with AD and cognitively healthy (CH) older adults, and reproduced the HMM-based segmentation of the regional electrophysiological fluctuations into a set of recurring transient states with network topographies similar to those previously described (Baker et al., 2014). The temporal parameters of the HMM states provided unique information about the short timescale abnormalities of the oscillatory activity in the functional networks in AD. Additionally, to better understand how these fast electrophysiological states may relate to the prior fMRI findings, we explored the slow timescale structure of the occurrence rates of the HMM states in CH older adults. The results demonstrate that MEG-estimated fast states of intrinsic network synchrony differ substantially between patients with AD and CH individuals, and may provide a valuable marker of AD pathologic processes to be utilized in future studies.

## 2 Methods

### 2.1 Participants

10 patients with AD and 10 CH individuals were enrolled in this study through the Brain Aging and Dementia (BAnD) Laboratory at Massachusetts General Hospital (MGH), from a local longitudinal cohort or through community outreach. Participants were also referred to the study through the MGH Alzheimer's Disease Research Center (MGH ADRC). CH individuals were non-demented with Mini-Mental State Examination (MMSE) scores >26. All participants in the patient group were referred to the study with the clinical diagnosis of Alzheimer's disease. Participants were excluded for significant health concerns outside of the domains of study that would prevent participation or would be likely to confound study results. These conditions included major neurological or psychiatric disorders (e.g. Parkinson's disease, Huntington's disease, vascular dementia, clinical stroke, brain surgery, psychosis, severe major depression, moderate to severe traumatic brain injury), or any substantial systemic illness. All individuals had at least a high school education (12 years). The Partners Healthcare institutional review board (IRB) approved this work and informed consent was obtained from each participant. The participant groups were comparable in age, sex distribution, and years of education. Their demographic characteristics and scores on the cognitive/functional assessment are provided in Table 1.

**Table 1:** Demographic and clinical characteristics of study participants.

Assessment	AD	CH	*p*-Value
Age	76.65 ± 8.99	76.26 ± 12.08	n.s
Gender	6 males	5 males	n.s
Years of education	15.00 ± 2.87	16.00 ± 1.63	n.s.
MMSE	21.40 ± 4.62	28.30 ± 1.42	<0.001
MoCA	17.20 ± 5.22	27.10 ± 2.88	<0.0001

Note: Shown are means and standard deviations. Abbreviations: AD, Alzheimer's disease, CH, cognitively healthy, MMSE, Mini-Mental State Examination, MoCA, Montreal Cognitive Assessment, n.s., not significant.

### 2.2 Data acquisition

MEG data were acquired while participants were seated inside a magnetically shielded room (IMEDCO). Participants were instructed to keep their eyes on a fixation dot displayed in the center of the screen approximately 1 m in front of them. Three consecutive 3 min long recordings (filter, 0.03–330 Hz; sampling rate, 1000 Hz) were obtained using a whole-head VectorView MEG system (Elekta-Neuromag; 306 sensors arranged in 102 triplets of two orthogonal planar gradiometers and one magnetometer). The position of a participant's head with respect to the MEG sensor array was recorded continuously by means of four head position indicator coils (Uutela et al., 2001). To enable co-registration of the MEG and MRI data, a Fastrak digitizer (Polhemus), integrated with the Vectorview system, was used to record the locations of three fiduciary points (nasion and auricular points, which defined a head-based coordinate system), as well as of the four head position indicator coils and approximately 200 points, which digitized the head surface. The electrooculogram was recorded concurrently to identify epochs containing artifacts due to vertical and horizontal eye movements or blinks.

Structural MRI data (T1-weighted magnetization-prepared rapid gradient echo, [MPRAGE]; voxel size, 1 mm^3^; FoV, 256 × 256 × 176; repetition time 2530 ms; echo time, 1.64 ms; inversion time, 1200 ms; flip angle, 7°) were acquired using a 3.0T Siemens Trio whole-body magnetic resonance scanner (Siemens Medical Systems) and a 32-channel head coil. Structural images of each participant were registered to the MNI152 standard brain to allow performing all subsequent source space analyses in the MNI space. The locations of the MEG sensors with respect to the anatomy were determined by registering the head surface digitized during the MEG session to the head surface extracted from the structural MRI.

### 2.3 Data pre-processing

Each MEG recording was visually inspected to identify channels and/or time-intervals of data containing obvious artifacts (e.g., extremely high variance), which were discarded. The data were spatially filtered using the signal space separation method (Elekta-Neuromag Maxfilter software) to suppress noise generated by sources outside the brain and correct for head motion (Taulu et al., 2004; Taulu and Simola, 2006). Cardiac and ocular artifacts were removed by signal space projection (Uusitalo and Ilmoniemi, 1997), using the MNE-python software (Gramfort et al., 2013; Gramfort et al., 2014). Following artifact rejection, the MEG data were converted to SPM12 (Friston et al., 2007), and frequency filtered into a wide band between 4 and 30 Hz, which is characterized by a relatively high signal to noise ratio (SNR) (Hipp and Siegel, 2015). Because neural populations within different functional brain networks have previously been shown to oscillate at slightly different frequencies (Hacker et al., 2017; Mantini et al., 2007), three additional datasets were created by frequency filtering the data into narrow bands, including theta (4–7 Hz), alpha (8–12 Hz), and beta (15–30 Hz). In addition, to quantify ultra-slow electrophysiological potentials (see Appendix D), we lowpass-filtered the data at 0.1 Hz. All datasets were then downsampled to 200 Hz.

### 2.4 Analysis tools

MATLAB (The MathWorks Inc., Natick, MA) scripts utilizing several software packages were used to analyze the data (see https://www.ohba.ox.ac.uk/groups/analysis-group for sample scripts). The software packages included FSL (Jenkinson et al., 2012), SPM12 (Friston et al., 2007), Fieldtrip (Oostenveld et al., 2011), and FreeSurfer (Dale et al., 1999; Fischl et al., 1999).

### 2.5 Source analysis

The pre-processed MEG data, wide/narrow band and ultra-slow datasets, were projected separately onto a regular 8-mm grid spanning the entire brain using a linearly constrained minimum variance (LCMV) scalar beamformer implemented in SPM12 (Van Veen et al., 1997; Vrba and Robinson, 2001; Woolrich et al., 2011). Previously, beamforming has been shown to provide verifiable neural source estimates and to effectively reject interference from non-brain sources in the MEG signal (Sekihara et al., 2006; Sekihara et al., 2001). To account for variations in the sensitivity of the beamformer at different locations in the brain, the projected data were scaled by an estimate of the projected noise (Van Veen et al., 1997; Vrba and Robinson, 2001).

Following beamformer projection, time-courses from 38 anatomical regions of interest (ROIs) were prepared for the network analysis as previously described (Colclough et al., 2015, Colclough et al., 2016; O'Neill et al., 2015). The ROIs were chosen based on a group spatial independent component analysis (ICA) of fMRI resting recordings in the first 200 participants in the Human Connectome Project database (Van Essen et al., 2013). This same set of ROIs has been used previously to analyze functional networks based on MEG resting scan data (Colclough et al., 2015, Colclough et al., 2016). Regional time-courses were obtained based on the projected wide-band MEG data using a principal component analysis (PCA) of the voxel time-courses within each ROI, normalized so that the positive peak had a height of unity in all regions. The time-course for an ROI was represented by the coefficients of the (first) principal component accounting for most variance, weighted by the strength of the ICA spatial map. Oscillatory activity with zero-phase-lag, which likely contains “signal/spatial leakage” to multiple MEG sensors and may lead to inflated connectivity estimates, was accounted for by symmetrically orthogonalizing all ROI time-courses simultaneously (Colclough et al., 2015). The multivariate symmetric orthogonalization (Everson, 1999; Löwdin, 1950) produces a unique solution that is unaffected by any re-ordering of ROIs and constitutes an optimal set of mutually orthogonal ROI time-courses, which are minimally displaced from the uncorrected original time-courses (as measured by the least-squares distance). By being multivariate, this method can also account for any spurious associations inherited from true connections (Colclough et al., 2015).

Following leakage reduction, the amplitude envelope of the oscillatory wide-band activity for each ROI time-course was derived by taking the absolute value of its Hilbert transform and downsampling to 20 Hz (Kiebel et al., 2005). These time-courses of oscillatory amplitude fluctuations were demeaned and normalized by the global (over all voxels) variance, and then, concatenated temporally across all participants.

To allow for the computation of spatial maps at higher spatial resolution, oscillatory amplitude time-courses were also obtained from each brain voxel, separately for the wide-band and each of the narrow-band datasets. Following beamformer projection, the amplitude envelopes were computed as the absolute value of Hilbert-transformed raw data and downsampled to 20 Hz. Following beamformer projection, the ultra-slow data was also downsampled to 20 Hz.

### 2.6 Hidden Markov model

The HMM inference was conducted using previously described computations (for exhaustive details, see Baker et al., 2014). An HMM with 10 states was inferred based on the time-courses of the wide-band oscillatory amplitudes from 38 ROIs, concatenated across all participants. HMMs with 8 and 12 states were also inferred based on the data concatenated across all participants and yielded similar core results (presented in the Appendix A). The model with 10 states was selected because it produced a clear-cut replication in our older CH sample of the spatial patterns of HMM states previously described in younger participants (Baker et al., 2014). Furthermore, in an earlier HMM analysis that compared models with 3–12 states, free energy estimates reached the floor effect at 10 states (Rukat et al., 2016). For the model with 10 states, we replicated similar core results in group-specific HMMs inferred separately in the datasets concatenated across the CH or AD participants (presented in the Appendix B). To illustrate utility of the HMM in describing the network dynamics, we compared the 10-state HMMs between the real data and 100 datasets simulated from random normal deviates, which were of the same dimensionality and were similar to the real data in frequency spectra and inter-timeseries correlations. The HMM with relatively simple state observation models (Baker et al., 2014) parsed the rich temporal dynamics of the surrogate datasets into separate states. Nonetheless, the temporal parameters of the HMM states inferred in each surrogate dataset, which was stationary by design, lacked robust profiles characteristic of the real data (presented in the Appendix C).

**Fig. 1:**
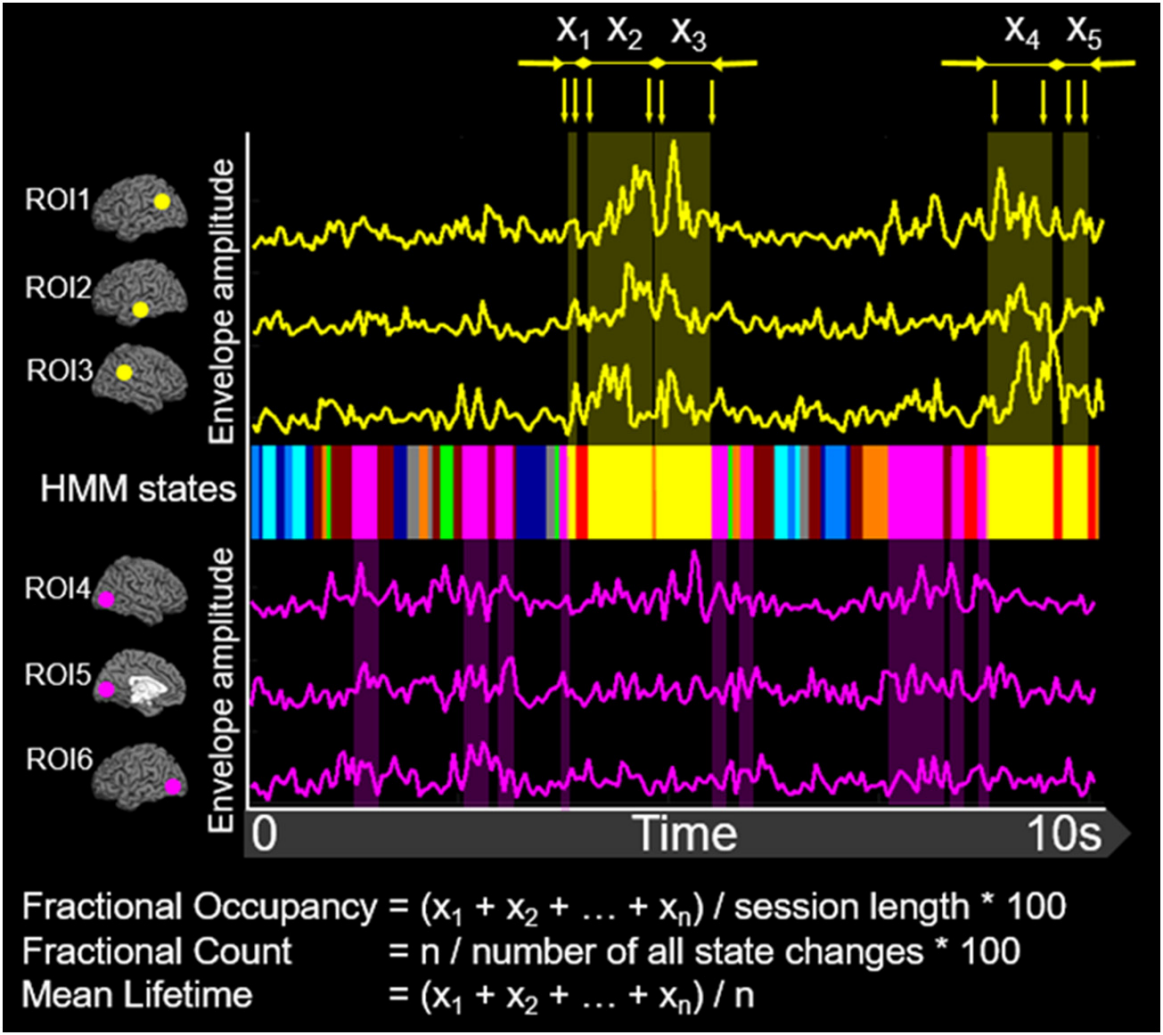
Sample data obtained in a CH participant during a 10s-long time-interval. A multicolored band in the center shows a sequence of network states inferred by the HMM; 10 different colors correspond to 10 states characterized by distinct spatial patterns of the brain oscillatory activity. Note, the timing of the HMM state shown in yellow coincides with increases in the envelope amplitude time-courses shown in yellow in the upper pane. It so happens that all these time-courses were extracted from the known nodes of the DMN, as is shown on the top/left (loci in the cerebral cortex are represented by yellow dots). In contrast, visits to the HMM state shown in magenta are temporally concordant with rises in the envelope amplitude time-courses shown in magenta in the lower pane. It so happens that these time-courses were extracted from the known nodes of the VisN, as is shown in the lower left (loci are represented by magenta dots). Several temporal metrics of the inferred HMM states can be computed. For instance, given that the yellow state (labeled DMN) was visited *n* number of times during the recording session, and the visits lasted for *x*_*1*_, *x*_*2*_, … *x*_*n*_ ms, fractional occupancy, fractional count, and mean lifetime can be computed for this state as shown in the bottom.

Fig. 1 illustrates the basic principles of the HMM inference; the model detects transient states when coordinated changes in the oscillatory amplitude recur in a distinct set of brain regions, and infers a time-course for each state. The individual HMM states are defined by a unique multivariate normal distribution over the ROIs (a mean vector [M × 1] and a covariance matrix [M × M], where M = 38, the number of ROIs; Rezek and Roberts, 2005). To account for variability in the HMM inference due to different initializations, 10 realizations were performed and the model with the lowest free energy was selected. The Viterbi algorithm was applied to identify the most probable state at a given time point (Rezek and Roberts, 2005). Time-courses for each state were constructed as indicator variables specifying the time intervals when the state is most probable.

### 2.7 State spatial maps

Following Baker et al. (2014), anatomical regions exhibiting state-specific activity (i.e., changes in oscillatory amplitudes during an HMM state, relative to what is happening on average over time) were mapped by computing partial correlations within the general linear modeling (GLM) framework. Several types of maps were derived. First, to determine which of the 38 a priori ROIs show activity changes during each network state, partial correlations were computed using the time-courses of the HMM states and the time-courses of the wide-band oscillatory amplitudes in each ROI. While the HMM was inferred on ROI time-courses in order to reduce the dimensionality of the data to a computationally manageable amount, this did somewhat limit the potential spatial resolution. Therefore, to map state-specific activity within the ROIs with higher resolution, we also calculated the partial correlations of the HMM state time-courses using the wide-band amplitude envelope at each brain voxel. An additional map was created based on correlations of each HMM state time-course with the wide-band amplitude envelope at each brain voxel that were computed selectively within time-intervals of the high occurrence rate of the state (presented in the Appendix D, Fig. D.3). Finally, to map frequency-specific activity during HMM states, the partial correlations of the HMM state time-courses were computed with the narrow-band amplitude envelope at each voxel separately for the theta, alpha, and beta frequency bands.

A similar approach was employed to map the regions where the time-courses of the ultra-slow electrophysiological potentials correlate with the occurrence rates of the fast HMM states. The long timescale time-course of the occurrence rate fluctuations for each HMM state was quantified as changes in the proportion of time spent in the state within 5-sec-long sliding windows (half a cycle length in 0.1 Hz oscillation). Partial correlations were computed between these state-rate time-courses and the lowpass filtered (<0.1 Hz) electrophysiological signal fluctuations at each voxel (the analysis of the long timescale structure of the HMM states is presented in the Appendix D).

In all GLM analyses conducted to compute the cortical maps, we employed a design matrix (T × K), where K is the number of states and each of the K columns is a state time-course with T time-points (Brookes et al., 2004; Friston et al., 1996). For each participant, at each ROI/voxel, a multiple linear regression was performed with the time-course of the electrophysiological activity as the dependent variable. Prior to fitting the GLM, both the design matrix and the ROI/voxel data were normalized to have zero mean and unit variance. Estimates of the partial correlation coefficients between each state and the ROI/voxel data yielded a set of K spatial maps. These maps were averaged across participants and visualized on the cerebral cortex. In the ultra-slow analysis, averaging across participants was based on the absolute values of the coefficients to account for the ± π phase ambiguity of MEG-based estimates of the cortical activity, which are influenced by the arbitrary default orientation of elementary current dipole sources constrained to the local cortical anatomy (Baillet et al., 2001).

### 2.8 A spatial map collapsed across a subset of HMM states

**Fig. 2:**
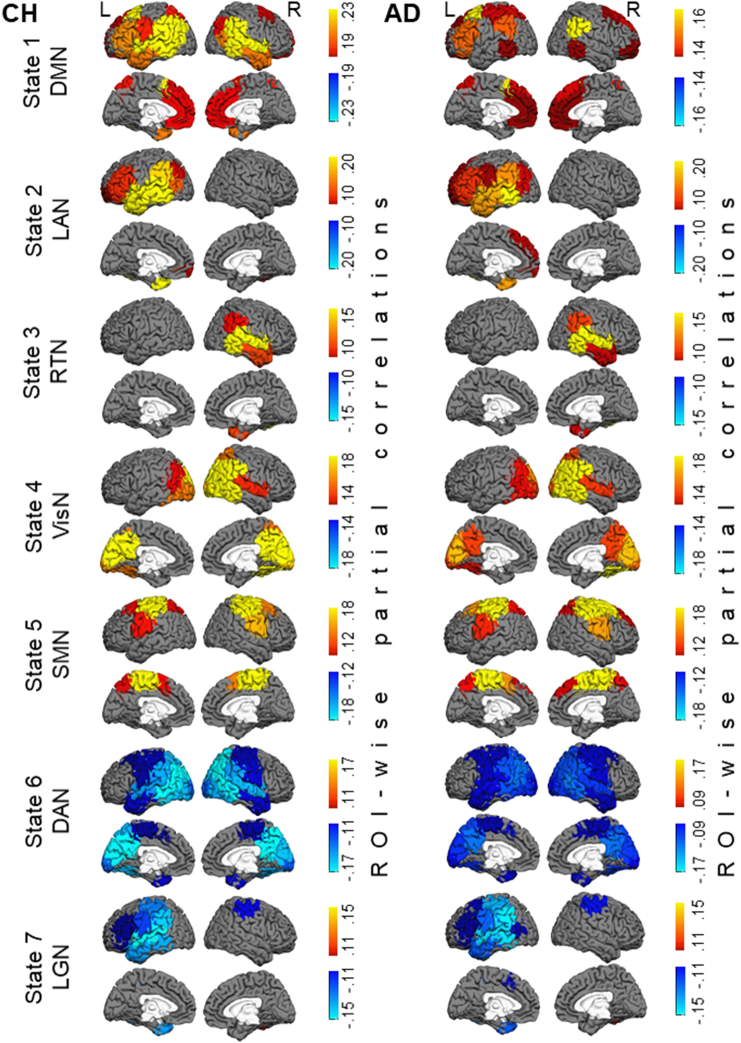
Maps of state-specific increases (in yellow/red/brown colors) and decreases (in blue color) in oscillatory amplitudes during seven salient network states inferred by the HMM. Each map shows partial correlations between the state time-course and the oscillatory amplitude envelopes in a priori ROIs. Maps in the CH group are shown in the left panel and in the AD group in the right panel. Note, for States 1 & 6, the maps are shown using different color scales for the CH and AD groups to optimally illustrate the network topography in each group. Abbreviations: CH, cognitively healthy, AD, Alzheimer's disease, DMN, default mode network, LAN, left associative network, RTN, right temporal network, VisN, visual network, SMN, sensorimotor network, DAN, dorsal attention network, LGN, language network.

**Fig. D.2:**
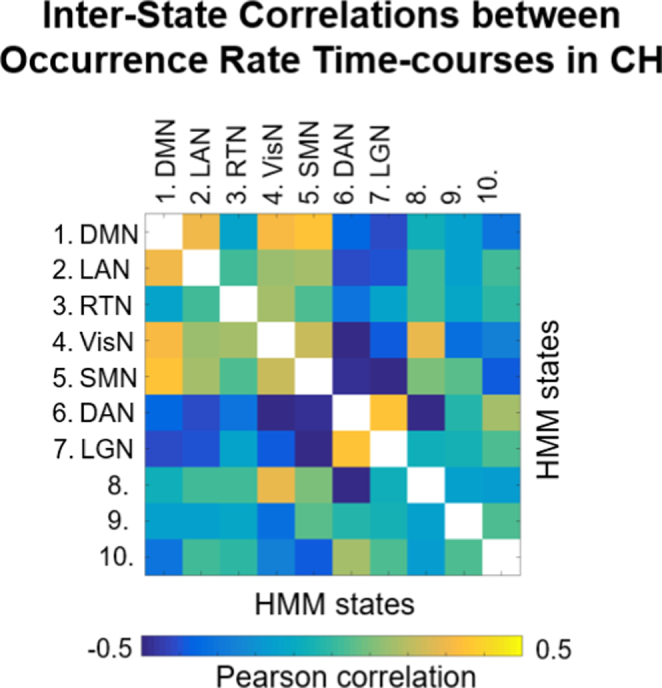
Temporal relationships between visits to different HMM states. Fluctuations in the rate of occurrence of each HMM state over time were quantified as changes in the proportion of time spent in the state within 10-sec-long (following Baker et al., 2014) sliding windows. Shown are Pearson correlations between the occurrence rate time-courses in each pair of states. This pattern of inter-state temporal relationships is similar to that in prior HMM on MEG data (Baker et al., 2014). Abbreviations are the same as in Fig. 2.

**Fig. D.3:**
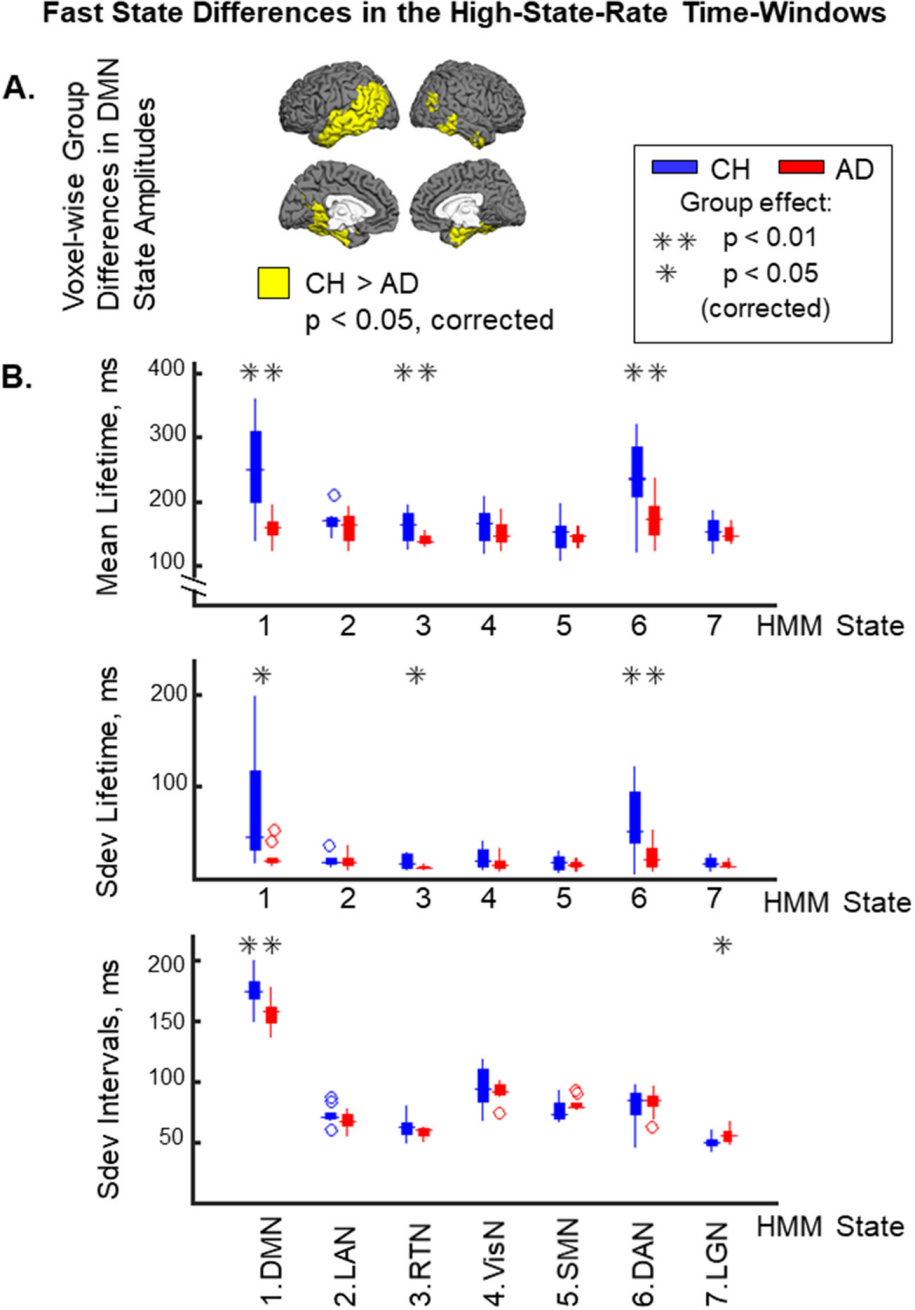
Results selectively within the time-intervals exhibiting increased recurrence rates of individual HMM states. A. Maps of group differences in the voxel-wise activity increase specific to the transient DMN state visits during the time-intervals when the DMN-state rate of occurrence was high. Brain voxels where the state-specific enhancement in the oscillatory amplitude was reduced in the AD relative to CH group are shown in yellow color. B. Group differences in temporal characteristics of the inferred fast states. Boxplots illustrate value distributions (as described in Fig. 6) of mean lifetime (top), standard deviation of lifetimes (middle), and standard deviation of inter-state intervals (bottom) for seven salient states (labeled in the bottom). Abbreviations are the same as in Fig. 2.

In the earlier HMM of the MEG data, weak partial correlations were observed between the activity in the precuneus/PCC and the time-course of coordinated activity changes in regions overlapping other DMN nodes (Baker et al., 2014). In part, this result could be due to relative insensitivity of MEG to deep sources, such as those on the medial cortex. Notably, the precuneus/PCC has been previously found to act as a functional connectivity “hub” (Buckner et al., 2009; Deco et al., 2017a; Deco et al., 2017b; Sporns et al., 2007), and might have been active during periods of enhanced activity in several networks (i.e., during several HMM states). To further characterize the precuneus/PCC engagement during the HMM states, the present study obtained voxel-wise maps of consistent activity changes, relative to what is happening on average over time, during a set of time-windows encompassing the DMN, VisN, SMN, and left associative network (LAN) states. These four networks were selected as likely candidates for cross-network interaction with the DMN because [1] in the earlier HMM (Baker et al., 2014), activity states in similar networks exhibited fluctuations in the rate of occurrence over time that correlated with the occurrence rate of the DMN (confirmed in our CH sample, see Appendix D, Fig. D.2), and showed relatively high probabilities of inter-states transitions to/from the DMN (confirmed in our CH sample, see Fig. 7), [2] during the time-intervals of heightened occurrence rates of the DMN state, when the oscillatory amplitudes in the DMN nodes were internally strongly inter-correlated, the functional networks similar to the selected four displayed high correlations in their oscillatory amplitudes with the PCC (Baker et al., 2014; de Pasquale et al., 2012). For the GLM analysis, the original 10-state design matrix (T × 10; T = number of time-points) was modified by collapsing four columns, corresponding to the DMN, VisN, SMN, and LAN time-courses, into one. The resulting design matrix (T × 7) included one column with the combined multi-state time-course of T time-points (indicating time-periods when DMN, VisN, SMN, or LAN were the most probable state), and 6 remaining columns of T time-points each, including time-courses of other HMM states, which were unchanged.

### 2.9 Maps of between-group effects

To examine differences in the HMM state spatial maps between the AD and CH groups, coefficients obtained in the multiple regression of the HMM state time-courses on the amplitude envelope in each brain voxel were compared using an independent samples *t*-test. To assist the comparisons with other studies, maps of oscillatory amplitudes in three frequency bands (theta, alpha, beta) averaged across the entire recording of each participant were also compared between the study groups using an independent samples *t*-test. To correct for multiple comparisons, the family-wise error rate was computed using Threshold-Free Cluster Enhancement in FSL Randomise software. The group difference maps were visualized on the cortical surface using FreeSurfer software. Group differences in the inter-participant variance were examined using a Bartlett test.

### 2.10 Metrics of network dynamics

To quantify the temporal characteristics of the inferred HMM states, we obtained a number of summary metrics (see Fig. 1 & Baker et al., 2014). For the purpose of computing these metrics, the states were classified as being on or off by choosing the most probable state at each time point (i.e. the Viterbi path). *Fractional occupancy* is defined as the fraction of the overall recording time spent in each state. *Fractional count* is defined as the number of times each state is visited as a fraction of the total number of state transitions. *The mean lifetime* is defined as the average amount of time spent in each state before transitioning out of that state. *Transition probability* is defined as the probability of transitioning to any particular state given the current state.

To examine differences in these metrics between the AD and CH groups, independent samples t-statistics were computed. Statistical significance of any findings was tested by random permutation of the group labels. Group differences in the inter-participant variance were examined using a Bartlett test.

## 3 Results

This section presents the results of our primary analysis (several additional analyses are presented in the Appendix). An HMM with 10 states was inferred based on estimates of neural activity fluctuations during a resting scan, which were obtained from source-localized MEG of all study participants that was co-registered to a common brain template. The inferred HMM states represent distinctive spatiotemporal patterns of coordinated wide-band (4–30 Hz) oscillatory changes that recurred at different points in time within specific neural networks. In their cortical topographies, the detected networks were comparable to those previously described based on MEG, using similar acquisition and analysis methods (Baker et al., 2014), and overlapped well-established functional networks that exhibit spontaneous regional correlations measured by fMRI (Biswal et al., 1995; Corbetta and Shulman, 2002; Di and Biswal, 2014; Fox et al., 2006; Greicius et al., 2003; Lowe et al., 1998).

**Fig. 3:**
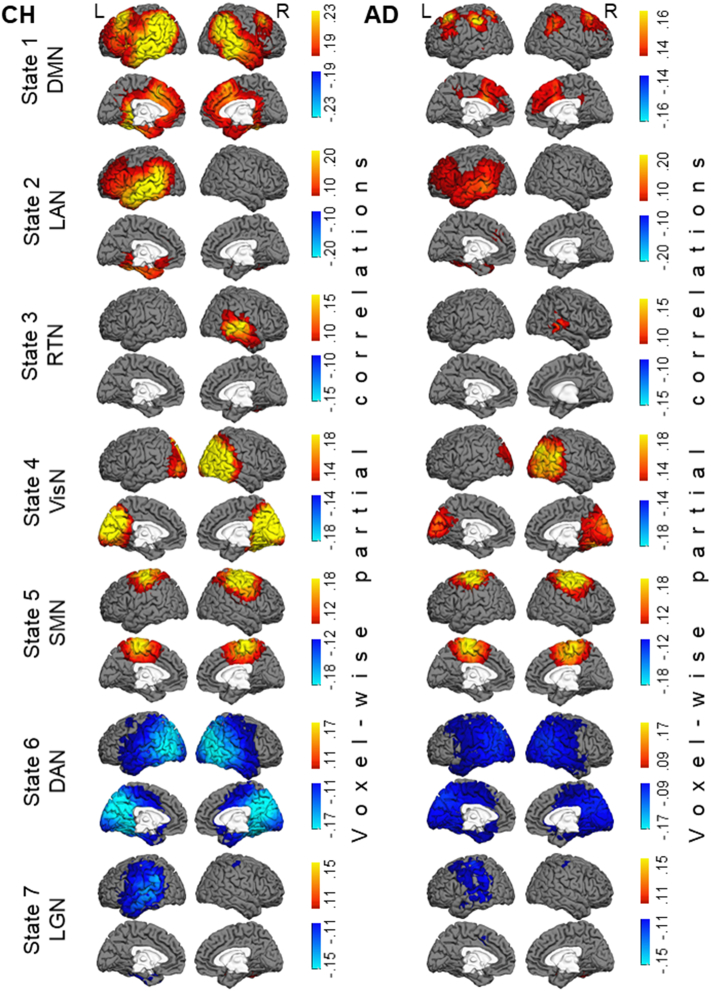
Maps of seven salient HMM states at the brain voxel resolution level. Maps of state-specific increases (in yellow/red/brown colors) and decreases (in blue color) in oscillatory amplitudes in the CH group are shown in the left panel and in the AD group in the right panel. Each map shows partial correlations between the state time-course and the oscillatory amplitude envelopes in individual brain voxels. Color scales and abbreviations are the same as in Fig. 2.

**Fig. 4:**
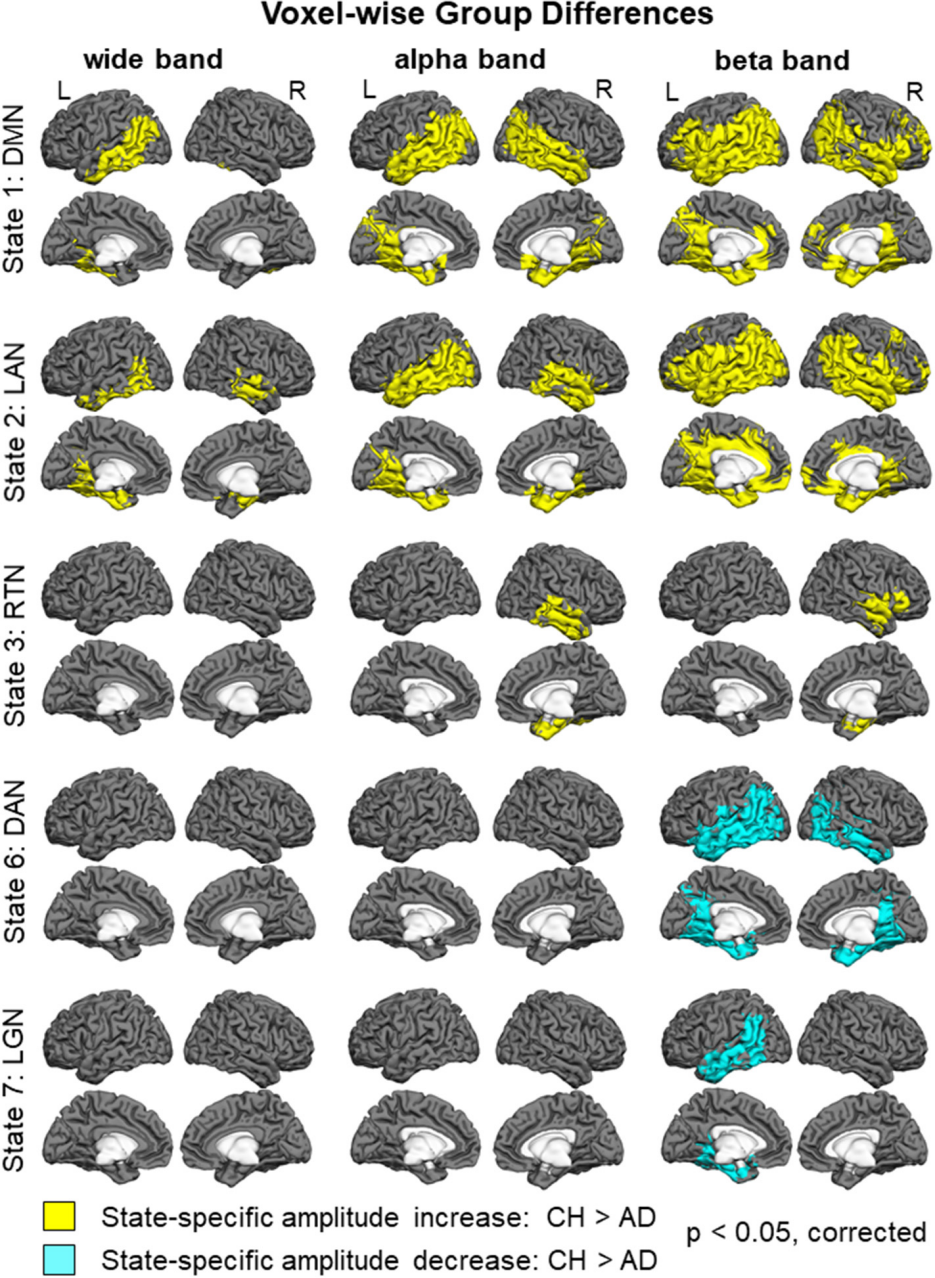
Maps of group differences in the voxel-wise state-specific activity changes in the wide band (4–30 Hz) in the left panel, in the alpha band (8–12 Hz) in the central panel, and in the beta band (15–30 Hz) in the right panel. Brain voxels where the state-specific enhancement in the oscillatory amplitude was less prominent in the AD relative to CH group are shown in yellow color. Brain voxels where the state-specific attenuation in the oscillatory amplitude was less prominent in the AD relative to CH group are shown in blue color. Abbreviations are the same as in Fig. 2.

The HMM was inferred from the oscillatory amplitudes of group-concatenated data that included both CH individuals and patients with AD. Following the model inference, the partial correlation maps, indicating where in the brain there are relatively high or low amplitudes in each state, compared to what is happening on average over time, were computed for each individual participant, and the maps were averaged across each participant group. Fig. 2 displays the ROI-wise maps of state-specific changes in wide-band oscillatory amplitudes in seven salient networks, derived in the HMM analysis. The maps show the partial correlations between a state time-course and the oscillatory amplitude envelopes in a priori ROIs. We supplemented the ROI-wise maps, which were limited in spatial resolution, with voxel-wise maps that are displayed in Fig. 3. These maps show the partial correlations between a state time-course and the oscillatory amplitude envelope in individual brain voxels. Because very few topographical differences were evident between the maps in Fig. 2, Fig. 3, we examined group differences in the state-specific activity using the maps with the higher spatial resolution. Fig. 4 shows differences between the AD and CH groups at the brain-voxel resolution level for the wide-band, alpha-band, and beta-band data.

In CH individuals, State 1 was characterized by enhanced oscillatory amplitudes in the regions overlapping the DMN, including bilateral inferior parietal lobe, medial prefrontal cortex, and lateral temporal cortices (Biswal et al., 1995; Di and Biswal, 2014; Greicius et al., 2003). Similar to prior MEG results (Baker et al., 2014), no robust effect was observed in the precuneus/PCC region. The maps of this state (which, following Baker et al. (2014), we labeled as DMN) showed prominent differences between the AD and CH groups; the state-specific effect in AD was much smaller in the inferior parietal cortex and the lateral temporal cortex, particularly in the left hemisphere (note, the color scales of the displayed correlations in Fig. 2, Fig. 3 are different between the groups). State 2 in CH participants was characterized by a left-lateralized increase in oscillatory amplitudes within the associative cortex of the prefrontal, temporal, and inferior parietal lobes. The map of state 2 (which we labeled as the left associative network, LAN), was also altered in AD; the effect was smaller in the temporal and inferior parietal regions.

No other states of the wide-band oscillations displayed significant differences in the brain topography between the AD and CH groups. State 3 (labeled as the right temporal network, RTN) showed a right lateralized enhancement in oscillatory amplitudes, relative to fluctuations over time on average, primarily in the temporal cortex. States 4 and 5 showed increased amplitudes in the bilateral visual network (VisN) and the sensorimotor network (SMN), respectively (Baker et al., 2014; Biswal et al., 1995; Lowe et al., 1998). State 6 was marked by a decrease in the oscillatory amplitude in the regions overlapping the dorsal attention network (DAN), including bilateral posterior lateral temporal cortex, intra-parietal sulcus, and extending toward the frontal eye fields (FEF) (Baker et al., 2014; Corbetta and Shulman, 2002; Fox et al., 2006; Fox et al., 2005). State 7 was characterized by an amplitude reduction in the language network (LGN) (Cordes et al., 2000; de Pasquale et al., 2012; Smith et al., 2012), including the left parietal-temporal and prefrontal regions (note, amplitude reductions in state 6 observed by Baker et al. (2014) had a similar topography). The time-courses of the remaining three states had weaker correlations with the regional fluctuations in oscillatory amplitudes (maps for these states are shown in the Appendix E). These states exhibited effects within subcomponents of some of the first 7 networks, and hence may constitute transitional states.

Group differences in oscillatory amplitudes were most prominent in the alpha (8–12 Hz) and beta (15–30 Hz) frequency bands. Both in the DMN (#1) and LAN (#2) states, the state-specific enhancement in alpha/beta amplitudes was lower in AD relative to CH in extensive bilateral regions primarily in the temporal and inferior parietal cortices. In the beta activity, the group effect was also observed in the lateral prefrontal cortex and precuneus, particularly in the left hemisphere. No significant group differences were observed in the theta band (4–7 Hz, *p* > 0.85 in all voxels, not shown). Notably, group differences selective to the higher frequency bands were observed in the RTN (#3), DAN (#6) and LGN (#7) states. During the RTN state, the enhancement in alpha/beta amplitudes was smaller in the AD than CH group in the right temporal cortex. In both DAN and LGN states, the state-specific reduction in oscillatory amplitudes in the parietal and temporal regions was less evident in the AD than CH group in the beta band. In the LGN, this effect was restricted to the left hemisphere. No frequency-specific group differences were found in the visual or sensorimotor cortices. When oscillatory amplitudes in the brain voxels were averaged across the entire session, no significant differences were found between the participant groups in any of the frequency bands (*p* > 0.1, in all voxels). For all comparisons, we confirmed that between-participant variability in the patient group could not account for the results. Variance in the data averaged across voxels within cortical nodes in the functional networks (Freesurfer parcellation, Yeo et al., 2011) in the AD group did not exceed that in the CH group (Bartlett test, no nodes with *p* < 0.1).

**Fig. 5:**
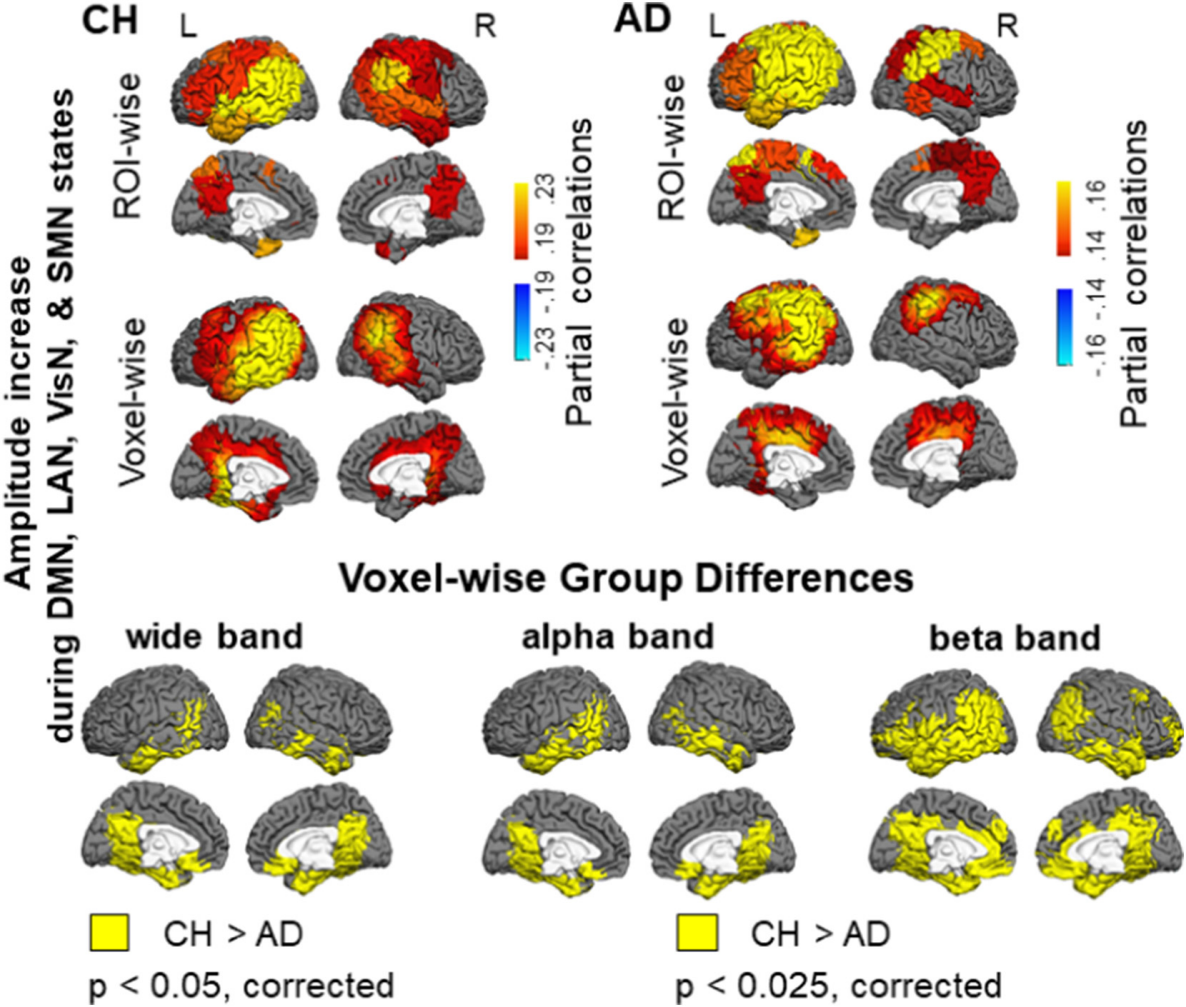
Maps of effects specific to a combined set of four HMM states, including the DMN, LAN, VisN, and SMN. ROI-wise maps on the very top show consistent enhancements (in yellow/red/brown colors) in oscillatory amplitudes during these network states, as quantified by partial correlations between the aggregate four-state time-course and the oscillatory amplitude envelopes in a priori ROIs. Directly below are the corresponding maps at the voxel-wise resolution, as quantified by partial correlations between the aggregate four-state time-course and the oscillatory amplitude envelopes in individual brain voxels. Maps in the CH group are on the left, and maps in the AD group are on the right. The bottom panel shows voxel-wise group differences. Brain voxels where the amplitude enhancement during the four network states was reduced in the AD relative to CH group are shown in yellow color in the wide band (4–30 Hz) in the left panel (*p* < 0.05, corrected), in the alpha oscillatory band (8–12 Hz) in the central panel (*p* < 0.025, corrected), and in the beta band (15–30 Hz) in the right panel (*p* < 0.025, corrected). Color scales and abbreviations are the same as in Fig. 2.

The precuneus/PCC region, which has been consistently linked with the DMN in the resting fMRI studies (Biswal et al., 1995; Di and Biswal, 2014; Greicius et al., 2003), was not a part of the DMN (#1) state both in the current (Fig. 2, Fig. 3) and previous HMM/MEG analyses (Baker et al., 2014). However, the oscillatory amplitude in the precuneus/PCC and several other DMN nodes in the CH group was consistently enhanced during a set of HMM states, including the DMN, LAN, VisN, and SMN, compared to what was happening on average over time. Fig. 5 shows the maps of partial correlations, averaged across the CH participants, between the combined time-course of these four HMM states and the amplitude fluctuations in the brain voxels. Corresponding maps for the AD group are also included, as well as the maps of group differences. In the AD patients, the activity increase during this four-state set was smaller in the precuneus/PCC and other regions overlapping the DMN, especially in the beta frequency band.

**Fig. 6:**
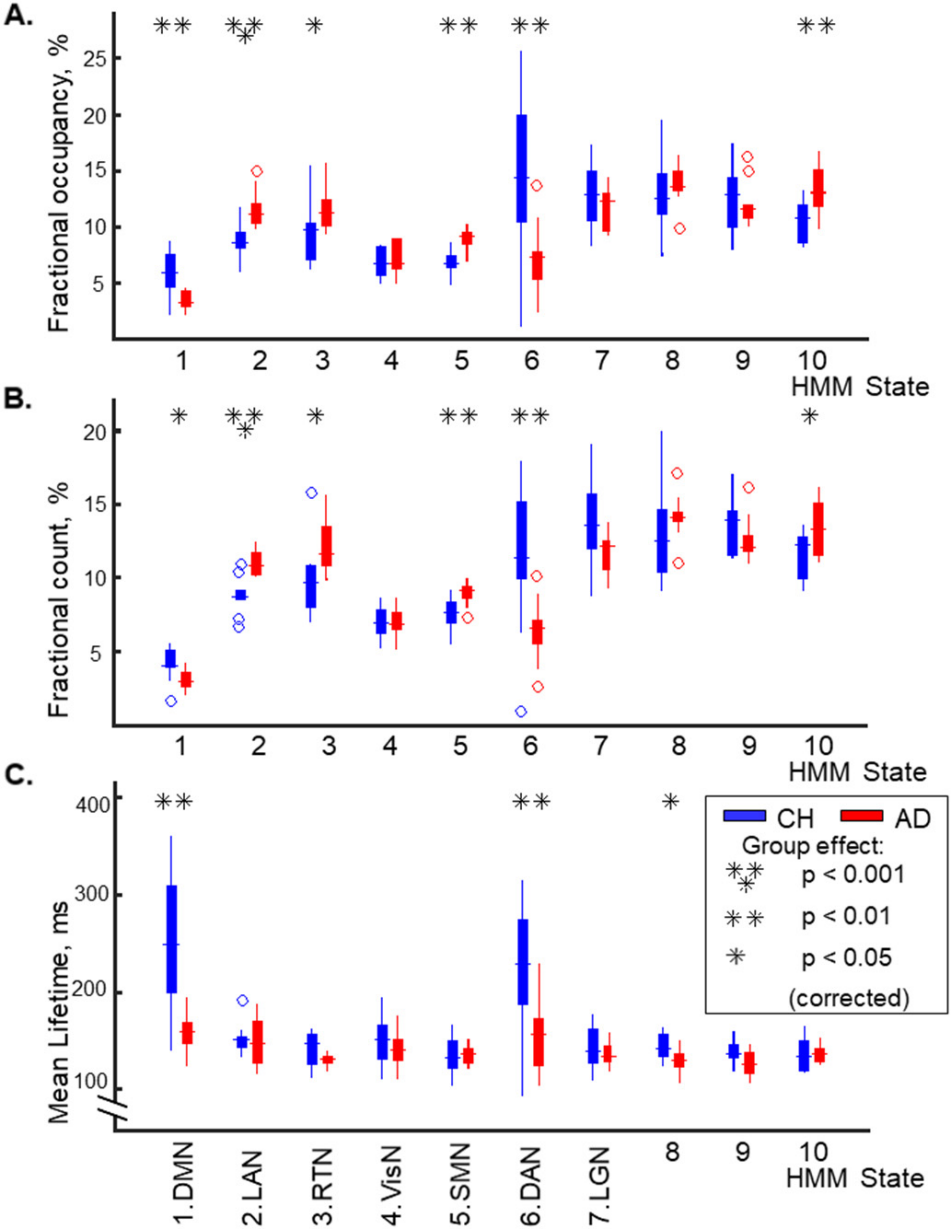
Group differences in temporal characteristics of the inferred HMM states. A. Fractional occupancy, or the proportion of time spent in each state. B. Fractional count, or the number of visits to each state as a proportion of the number of all state transitions. C. Mean lifetime, or the mean duration of the visits to each state. Boxplots for the seven salient states (1–7), labeled in the bottom, and for the three transitional states (8–10) show medians, 25th/75th percentiles, and outliers; whiskers extend to most extreme points but for outliers. Abbreviations are the same as in Fig. 2.

The HMM infers time-courses of visits to each of the brain states, revealing fluctuations in synchrony at timescales down to the order of hundreds of milliseconds. These state dynamics showed robust differences between AD and CH participants (Fig. 6). The overall temporal make-up of synchrony states in the brain activity, quantified by fractional occupancy, or the proportions of time the neural system spends in each spatial state of activity, showed pronounced differences between the groups (Fig. 6A). The proportion of time when the DMN (#1) and DAN (#6) states were visited was lower in the AD relative to CH group. Fractional occupancies for these states in the CH group (approximately, 6% & 14%, respectively) were comparable to those previously observed (Baker et al., 2014), but in the AD group, were only about half the magnitude (approximately, 3% & 7%, respectively). On the contrary, the fractional occupancies for two lateralized states (LAN #2 & RTN #3) and the SMN state (#5) were higher in the AD relative to CH group. Fig. 6B shows that, for the large part, group differences in the fractional occupancy could be accounted for by the fractional counts of state visits. The fractional count, or the number of visits to a state, normalized by the overall number of state shifts, was reduced for states 1 & 6 and increased for states 2, 3 & 5 in AD relative to CH participants. In addition, the mean lifetime, or the mean duration, of visits to the DMN (#1) and DAN (#6) states was shorter in the AD relative to CH group (Fig. 6C). All HMM states were on average short-lived in both groups, similar to previously observed (Baker et al., 2014). Mean lifetimes of most states lasted approximately 150 ms, but in the CH group, mean lifetimes of DMN and DAN states were longer than those of other states (*p* < 0.05, Bonferroni corrected), lasting between 200 and 250 ms. The mean lifetime for the DMN state in our older CH sample, matched to the AD patients in age, was slightly longer than that previously observed in younger participants (250 ms in our sample vs. 205 ms in Baker et al., 2014). In the AD group, the lifetimes for the DMN and DAN were considerably shorter (150 ms). For all comparisons, we confirmed that between-participant variability in the patient group could not account for the results. Variance in the HMM state parameters in the AD group did not exceed that in the CH group (Bartlett test, no effects with *p* < 0.1).

**Fig. 7:**
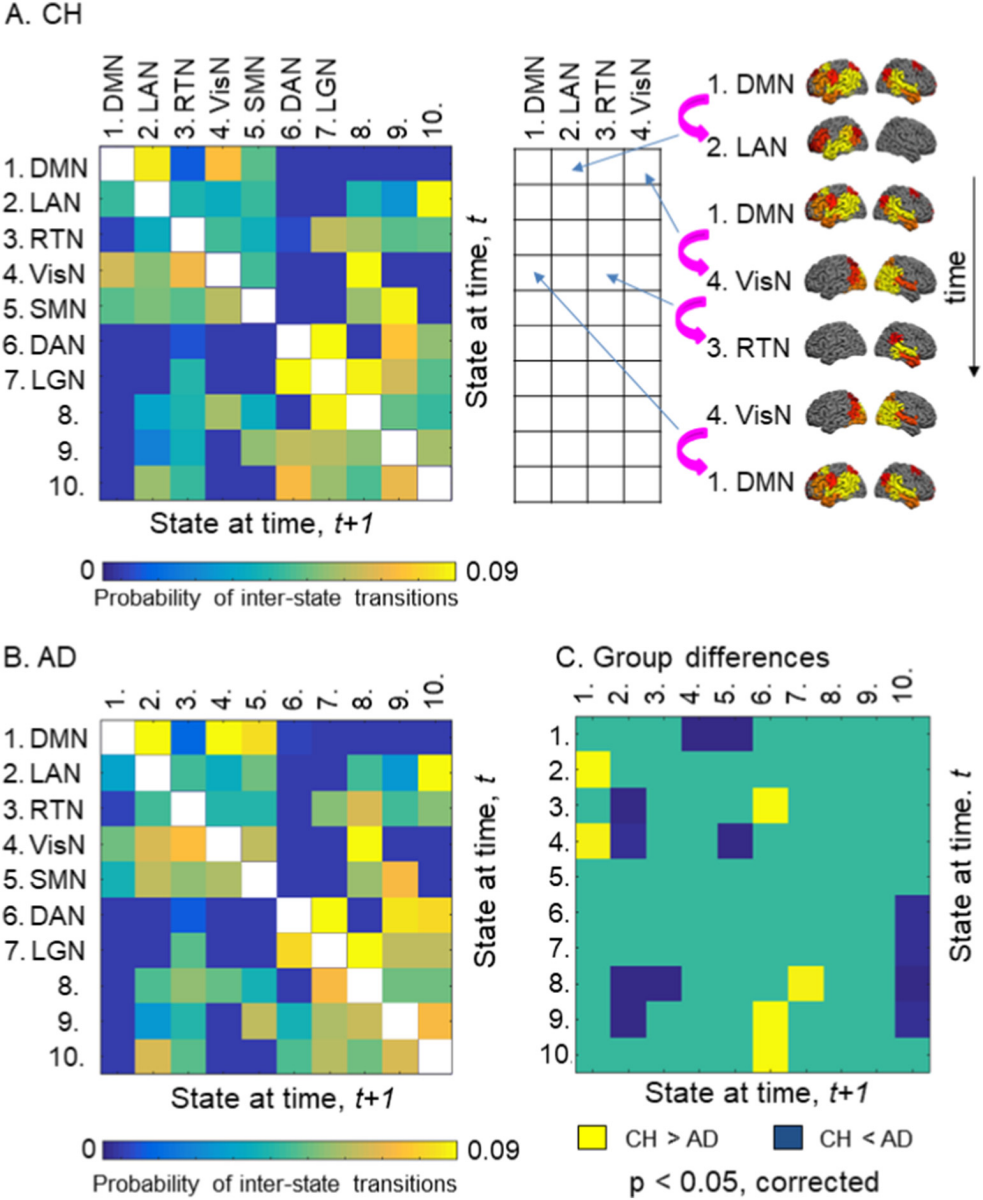
State-to-state transitions. A. Transition probability matrix for the CH group. Shown is the probability of transitioning from a certain state (labeled in rows) at the time-point, *t*, to any other state (labeled in columns) at the time-point, *t* + *1*. The correspondence between state-to-state transitions and the loci in the matrix is illustrated on the right. The transitions in a sample sequence of HMM states, indicated by magenta arrows, would be recorded within the first four columns of the matrix, as indicated by blue arrows. B. Transition probability matrix for the AD group. C. Group differences; the lower and higher transition probabilities in the AD group relative to CH group are represented by yellow and blue colors, respectively.

Patterns of transition between the activity states of individual networks also differed between the groups, as was evident from the estimated probabilities of shifting to any other state given the current state. Fig. 7A shows the probability of transitioning from a certain state (labeled in rows) at the time-point, *t*, to any other state (labeled in columns) at the time-point, *t* + *1*, for the CH group. Fig. 7B shows the transition matrix for the AD group. The structure of the matrices observed in both study groups was similar to that observed previously (Baker et al., 2014), suggesting certain regularities in the interactions between the functional networks. For instance, the probability of shifting between the DMN (#1) and the DAN (#6) states was very low, and the probability of transitioning from the DMN to the LAN (#2), VisN (#4), and SMN (#5) states was relatively high. Fig. 7C indicates whether the estimated probabilities of specific state-to-state transitions were different between the AD and CH groups. Interestingly, the LAN (#2) state was less likely to transition to the DMN (#1) state in AD individuals compared with CH, but the DMN was more likely to shift to the VisN (#4) and SMN (#5) states. Among the networks transitioning to the DMN (#1), the VisN (#4) had the highest probability in both groups. However, in AD patients, the probability of transition from the VisN to the DMN was reduced, but the probability of transition from the VisN to the LAN (#2) was higher. In fact, several other networks (States ##3, 8, 9) in AD transitioned more readily to the LAN (#2). For all comparisons, we confirmed that between-participant variability in the patient group could not account for the results. Variance in the transition probabilities in the AD group did not exceed that in the CH group (Bartlett test, no effects with *p* < 0.1).

## 4 Discussion

Fluctuations in MEG-based estimates of the electrophysiological activity in the brain during a resting scan were analyzed using a recently introduced methodology that can detect synchrony variations in the activity of cortical regions with high temporal resolution. The time-courses of the activity, estimated in 38 ROIs covering the entire cortex in patients with AD and older CH participants, were analyzed using an HMM to infer sequential transitions between ten underlying network states. These transient states represented distinct spatial patterns of intrinsic brain activity and lasted on average between 150 and 250 ms, recurring in a rapid succession. In older CH adults, the inferred states replicated key spatial and temporal parameters of the MEG-based states previously described using similar methodology (Baker et al., 2014), and in their spatial topography, showed correspondence to the classic large-scale functional networks (Biswal et al., 1995; Corbetta and Shulman, 2002; Di and Biswal, 2014; Fox et al., 2006; Greicius et al., 2003; Lowe et al., 1998). Many oscillatory and temporal properties of the inferred states were different in the AD group compared with the CH group. Particularly, the state of enhanced oscillatory amplitudes in the regions overlapping the DMN was visited less often and for shorter time intervals in the patients, indicating that spontaneous synchronization in this network is both less frequent and less stable in AD. At some of the DMN nodes, the visits to this state were also marked by smaller amplitude changes in AD compared with CH, especially in the higher frequency bands (alpha and beta), when precise timing of local neuronal firing would be essential to establishing oscillations (Buzsaki, 2006). This result underscores the relevance of MEG-based measurements of fast functional network states in the study of AD pathophysiology. Capacity to rapidly establish and flexibly update large-scale synchrony among the DMN regions may be critical for memory and other higher cognitive functions (Douw et al., 2015; Douw et al., 2016).

Our analysis yielded metrics that, because of their good spatiotemporal resolution, may prove sensitive indicators of the neural network health in future investigations. We note an arguably high statistical power of the HMM approach, as significant differences were detected between relatively small samples of AD and CH participants. Nonetheless, observations about the spatial patterns of source-localized MEG data should be interpreted with caution, owing to their inherent ambiguity due to the inverse problem and the limited spatial resolution of this signal (Dale and Sereno, 1993; Gross et al., 2013; Troebinger et al., 2014). Future research is needed to validate the spatial localization assumptions of the HMM electrophysiological states through electrocorticography or simultaneous EEG/fMRI recordings. Additionally, it will be important to replicate the findings and elucidate how the properties of fast neural network states relate to variables such as age and cognitive capacity by analyzing larger datasets; for example, an MEG dataset at the CamCAN repository includes over 600 cases, aged between 18 and 88, and currently offers demographic and cognitive characteristics, but in the future will also offer the genetic risk information (Shafto et al., 2014; Taylor et al., 2017) (available at http://www.mrc-cbu.cam.ac.uk/datasets/camcan/).

### 4.1 Transient states of spontaneous synchrony in large-scale functional networks in older adults

The HMM was applied to the oscillatory amplitudes in a concatenated dataset that included both CH individuals and patients with AD. The resulting clear correspondence between the inferred individual states across all participants afforded for a well-defined analysis at the group level. A limitation of this approach could be that the HMM would identify the spatiotemporal patterns that recur most consistently across both participant groups, potentially masking some group differences. Evidence against this possibility came from HMMs that were applied separately in the CH and AD groups and reproduced the patterns of the salient states (Appendix B). Notably, in neither the combined nor separate HMM analysis, was there any indication that the states inferred in the CH participants were biased toward an unexpected spatial pattern. A previous study, which used a comparable methodology to analyze the MEG data of healthy participants, noted that topographies of four most prominent states inferred by the HMM, regardless of the overall number of the inferred states (varied between 4 and 14), were similar to the classic large-scale functional networks, including the DMN, DAN, VisN, and SMN (Baker et al., 2014). In the present study, the maps of these four prominent states in the CH individuals bore close spatial resemblance to the MEG-based states inferred in that earlier study. Whether the observed group differences in the HMM state parameters represent a functional abnormality in AD or the underlying neurodegenerative effects is difficult to determine based on the present data. Future research must address this question, for instance, by studying the electrophysiological states in persons at risk for AD, and tracking the progression of the functional deficits in relation to the structural findings (e.g., local cortical thinning). Noteworthy, we found that inter-participant variability in the examined electrophysiological data was not increased in the AD relative to CH group. This, together with the stability of the map topographies in AD across the combined and group-specific HMM analyses, suggests that the detected patterns of the HMM state abnormalities reflect a prevalent attribute of the AD patients, rather than factors related to individual differences in neurodegeneration (and the resulting poor cross-patient correspondence in the MEG neural sources).

The temporal profiles of the HMM electrophysiological states were highly diverse, featuring distinct fractional occupancies and counts, transient lifetimes, and specific inter-state transition probabilities. In contrast, applications of the HMM to simulated multivariate timeseries with frequency spectra and inter-series correlations that were stationary over time (even though, across a dataset length, comparable to those of the real MEG-based time-courses) exhibited considerably less variation in the properties of the inferred states as well as more sluggish state time-courses (Appendix C). Because the temporal dynamics of each simulated time-course were complex relative to the simple state observation models employed by the HMM (Baker et al., 2014), it was not surprising that several states were inferred in the surrogate datasets. The striking discrepancy in the state parameters between the real and simulated data suggested that the HMM is sensitive to the physiological processes that may underlie the state-to-state transitions in the real data, but that were not modeled in the surrogate datasets. One factor potentially contributing to the temporal patterns of recurrence of different fast states in the real data could be endogenous fluctuations of cortical excitability within functional systems (He et al., 2008; Pan et al., 2013). Depolarizations of apical dendrites in cortical superficial layers (e.g., due to excitatory nonspecific thalamic inputs (Birbaumer et al., 1990; Mitzdorf, 1985)), by increasing excitability, might facilitate shifts to the network-specific fast activity states. Consistent with this possibility, we found that local ultra-slow electrophysiological potentials (<0.1 Hz), thought to reflect periodic cortical depolarizations (He et al., 2008; Pan et al., 2013), temporally correlated with the occurrence rates of HMM states, mainly, in the regions overlapping the corresponding maps of fast state-specific changes in the 4–30 Hz oscillatory amplitudes and the classic large-scale functional networks described by fMRI (Sporns, 2013) (Appendix D). As a rule, ambiguity in source modeling of the MEG data precludes drawing solid conclusions about spatial patterns of the brain activity (Dale and Sereno, 1993; Gross et al., 2013; Troebinger et al., 2014). Nonetheless, the observed association across both the temporal and spatial dimensions suggests a possible account of how the fast states may be related to fMRI measurements, especially given the previously established spatiotemporal links between the ultra-slow spontaneous fluctuations recorded by the fMRI and the high spatial resolution electrocorticography (He et al., 2008). It is possible that the slow timescale structure of both the fast electrophysiological states and the intrinsic BOLD signal variations is influenced by the cortical excitability.

The HMM analysis of time-courses from 38 a priori ROIs regressed out the zero-phase correlated signal, presumed to encompass artifact due to “signal leakage” to multiple MEG sensors (Colclough et al., 2015; O'Neill et al., 2015). Therefore, the obtained maps of the HMM states represent conservative synchrony estimates, with reduced likelihood of spurious partial correlations (Fig. 2). We supplemented the ROI-wise maps, which were limited in spatial resolution, with maps of voxel-wise state-specific effects, which were computed as partial correlations with unadjusted oscillatory amplitudes (Fig. 3). Remarkably, few differences were evident between the ROI-wise and voxel-wise results. Minor discrepancies primarily in the medial cerebral cortex might be due to increased susceptibility of these loci, buried deep inside the brain, to the signal leakage artifact.

Similar to the prior HMM on MEG (Baker et al., 2014), we observed two states characterized by the oscillatory amplitude enhancements that spatially overlapped with the DMN but were lateralized to the left and right hemispheres (LAN and RTN). The probability of transitioning between the DMN and LAN states, estimated by the HMM, was particularly high, suggesting that the two networks may be related. This finding agrees with a recent result from the whole-brain fMRI study that acquired the images with improved temporal resolution of 0.8 s and identified temporally-independent components of the DMN exhibiting lateralized, spatially-overlapping topographies (Smith et al., 2012). Interestingly, in our study the periods of activation of the DMN nodes also included the executive areas of the lateral prefrontal cortex, especially in the left hemisphere. This temporal overlap between the executive and the DMN states in our sample of older participants is consistent with prior fMRI studies that found reduced distinctions between the functional neural networks in older individuals (Ferreira et al., 2016; Keller et al., 2015; Ng et al., 2016).

Absence of a robust association between the activity fluctuations in the precuneus/PCC and the time-intervals of coordinated activity enhancement in regions overlapping other DMN nodes is in agreement with the prior HMM on MEG data (Baker et al., 2014). However, this finding is inconsistent with the classic view of the DMN coming from fMRI studies, which includes this region as a major network node (Buckner et al., 2008). Based on several mathematical models of the whole-brain activity, the living brain was recently suggested to be in a perpetual state of metastability that maximizes not only the functional segregation but also integration across the large-scale overlapping networks and across time (Deco et al., 2017a; Varoquaux et al., 2012). Evidence from structural and functional MRI studies indicated that the precuneus/PCC might perform as a ‘hub’ that both has extensive across-network connectivity (Buckner et al., 2009; Sporns et al., 2007) and supports binding of neural processes across time (Deco et al., 2017a; Deco et al., 2017b). Furthermore, in an MEG study, the amplitude of the oscillatory activity in the PCC during time-windows of the DMN internal synchronization showed the highest correlations with the node activity in other large-scale functional networks (de Pasquale et al., 2012). The present study examined whether, due to the ‘hub’ role, the precuneus/PCC may be consistently engaged during multiple states of network activity by computing partial correlations between fluctuations in the oscillatory amplitude in this region and the combined time-course of the DMN and three other HMM states. We expected that the VisN, SMN, and LAN would be most likely to interact with the DMN, because the probabilities of switching between their activity states and the DMN were relatively high, and fluctuations in their occurrence rate paralleled those of the DMN (Fig. 7A and Appendix D, Fig. D.2; also Baker et al., 2014). Voxels in the precuneus/PCC showed robust positive partial correlations with the aggregate time-course, assembled from the visits to the DMN, VisN, SMN, and LAN states, in line with the central role of this node in between-network functional interactions.

### 4.2 DMN neural oscillations in AD: Spontaneous synchronization deficit and its putative functional significance

Both in CH individuals and patients with AD, we isolated the transient time-windows during a resting scan when a coordinated increase in the amplitude of the electrophysiological oscillations, estimated from MEG data, was the most robust in the DMN regions. We then showed that visits to this DMN state in AD were shorter, occurred less frequently, and were characterized by less pronounced changes in the oscillatory amplitude, particularly in the associative cortex of the lateral parietal and temporal nodes. It has been argued that correlations among neuronal systems at smaller scales are likely to survive through the largest scales (Roberts et al., 2015). Therefore, while cognizant of the limitations of the MEG, which offers a large-scale activity measurement with inherently ambiguous source-localization (Dale and Sereno, 1993; Gross et al., 2013; Troebinger et al., 2014), we attempted to relate the present findings to the small-scale activity measurements in the animal literature, suggesting a provocative interpretation with potential clinical significance.

Oscillations are a manifestation of synchrony in neural firing (Buzsaki, 2006). Therefore, the oscillation deficit in AD may indicate underlying abnormalities in the neuronal architecture of the DMN that disrupt spontaneous synchronization of the neuronal discharges. Findings in animals suggest that synchronizing complex and distributed networks in the associative cerebral cortex may depend on bursting patterns of neuronal firing. In the associative cortex, as much as half of neuronal firing might occur in bursts, but in the sensory cortex, >80% of spikes occur in isolation (de Kock and Sakmann, 2008; Degenetais et al., 2002). Bursts optimized in length can deliver precise and reliable signals to the downstream neurons (Gabbiani et al., 1996; Otto et al., 1991). Further, neuronal bursts may be developmentally regulated, increasing in intensity and rate of occurrence with aging (Hickmott and Dinse, 2013; Kepecs and Lisman, 2003; Smith et al., 2000). Taken together, these observations indicate that the bursting firing patterns may promote stability in signaling that is needed for temporal organization of neuronal activity in the distributed associative networks, especially in the aging brain.

Recent findings in the AD animal model demonstrated that temporal parameters of the neuronal bursts may be critical in regulating both quality and quantity of released β-amyloid, the protein that accumulates in the brain of AD patients and is linked to neurodegeneration (Dolev et al., 2013). A tantalizing possibility is that the average duration of the HMM states during a resting scan might be a suitable proxy measure of the amount of bursting in the neural activity. The prolonged duration of state visits might reflect stability of the spatiotemporal activity patterns resulting from the neuronal bursts. Interestingly, two HMM states (DMN & DAN) that were marked by oscillatory amplitude changes in widespread associative regions showed the longest average durations both in the current CH group and the previous study (Baker et al., 2014). Moreover, the DMN state showed a disproportionate increase in the average duration in our sample of older HC participants relative to younger healthy participants in a previous study (Baker et al., 2014). Prior studies employing fMRI revealed that abnormalities in spontaneous DMN synchrony track with β-amyloid accumulation in the DMN regions during the AD progression, as determined by PET (Brier et al., 2014; Brier et al., 2012; Hedden et al., 2009; Schultz et al., 2017; Sheline et al., 2010b). In addition, some MEG-based connectivity measures, computed for the regions overlapping the DMN, based on phase relationships in the source-localized electrophysiological oscillations, showed correlations with β-amyloid levels in the cerebrospinal fluid both in mild cognitive impairment and Alzheimer's disease (Canuet et al., 2015; Yu et al., 2017). An interesting future direction will be to investigate whether abnormally low durations of the DMN state visits may be a sensitive predictor of the β-amyloid accumulation in the DMN regions. Better understanding of the DMN neural dynamics that may influence the dysregulation of the β-amyloid metabolism can potentially facilitate development of novel, alternative therapies for AD.

### 4.3 Relationship of the abnormalities in HMM states with prior MEG and EEG findings in AD

While generally consistent with prior studies of the resting MEG and EEG in AD, the present results reflect the ability of our new analytic approach to resolve the short timescale synchrony changes in the estimates of oscillatory activity localized to voxels of the brain. In previous investigations, magnetic or electric field measurements obtained and analyzed on the scalp in the sensor space were not readily interpretable with regard to the underlying neural network sources.

The spectral analysis of spontaneous oscillations, conducted in the sensor space over the entire recording, consistently detected a deficit in the higher frequency bands (alpha, beta). AD patients showed reductions both in the power of these oscillations recorded at individual sensors (Berendse et al., 2000; Besthorn et al., 1997; de Haan et al., 2008; Fernandez et al., 2006; Poza et al., 2007, Poza et al., 2008; Rodriguez et al., 1999), and in the synchrony metrics, such as the spectral coherence, phase lag index, and synchronization likelihood, which were computed between spatially separate sensors (Alonso et al., 2011; Cook and Leuchter, 1996; de Haan et al., 2009; Franciotti et al., 2006; Jelles et al., 2008; Leuchter et al., 1987; Locatelli et al., 1998; Stam et al., 2009; Stam et al., 2006; Stam et al., 2005; Stam et al., 2002; Wada et al., 1998). The present study substantially refined these prior observations in AD by estimating that the reduction in the alpha and beta amplitudes might occur primarily at the parietal and temporal nodes of the DMN, as well as for the beta band, at the executive regions of the lateral prefrontal cortex. Organization of synchronized neuronal discharges into rapid series, forming oscillations, may demand especially precise time-locking in the intricate networks of these associative regions (Buzsaki, 2006). Additionally, the HMM analysis discerned a distinct pattern of oscillatory abnormalities during a subset of brain states (DAN, LGN), when the normal attenuation of beta amplitudes, compared to what is happening on average over time, was less prominent in AD than CH participants. The neuronal mechanism of the decrease in oscillatory amplitudes during these states is yet to be understood, even if some clues come from prior observations that the hemodynamic response during a resting fMRI scan correlates with the alpha/beta amplitude positively in the DMN but negatively in the DAN (Mantini et al., 2007). The present result indicates that ability to detect oscillatory deficits can be improved if the opposite-polarity effects are disentangled in time and space. The HMM-based analysis revealed a pattern of robust alpha/beta amplitude abnormalities in AD relative to CH in the present participant sample, but voxel-wise group differences in neither alpha nor beta amplitudes, averaged across the entire recording session, reached statistical significance.

Another approach to analyzing the EEG field potentials in AD patients has focused on the short timescale changes in the scalp topography. Transient states of quasi-stable scalp topography, labeled ‘microstates’, in the healthy brain usually last for approximately 100 ms (Koenig et al., 2002). In AD patients, the microstates have been found to be abnormally short-lived, and had more anterior scalp topography (Dierks et al., 1997; Ihl et al., 1993; Stevens and Kircher, 1998; Strik et al., 1997). The aim of segmenting EEG into microstates based on changes in scalp topography is analogous to that in our study – to identify time-intervals when activity in distinct sets of neural nodes changes in amplitude. Nevertheless, the HMM approach previously has been shown to yield improved temporal distinctions between spatial states (Rukat et al., 2016). Furthermore, the microstates in the earlier studies of AD were not clustered into unique classes based on their scalp topography, thus leaving unanswered the question of whether the deficits may be limited to a certain scalp map(s) and the underlying specific functional neural network(s).

The present study estimated recurrent changes in the oscillatory activity in several unique neural networks. The results suggested that the patients' abnormalities in the state durations and the makeup of the activated nodes may be limited to a subset of the functional networks. Shorter state durations in AD patients were selective to the DMN and DAN HMM states. Furthermore, an abnormal distribution of the oscillatory amplitudes across the network nodes (reduced in the more posterior nodes) was observed primarily in the DMN and LAN HMM states. Between the DMN and LAN, which overlapped spatially, the temporal balance in AD patients was shifted toward the LAN – less distributed but with a relatively strong prefrontal/executive component. The DMN states in AD patients recurred at an aberrantly low rate, but the LAN showed the most robust abnormal increase in the rate of synchronizations. Additionally, there was a consistent reduction in the oscillatory amplitudes in the posterior DMN nodes during the DMN, LAN, VisN, and SMN states in AD, relative to CH, indicative of the suboptimal performance of these regions as hubs for inter-network interactions. This result was consistent with a previously reported deficit in AD in posterior connectivity hubs, observed both in the sensor space MEG data (reduced intramodular synchronization likelihood in the parietal hub; de Haan et al., 2012) and the source-localized MEG data (reduced hub metrics, based on phase lag index, for the parietal DMN regions within a multiplex network integrating five frequency-specific networks; Yu et al., 2017). The pattern of the state transition abnormalities in AD in the present study also suggested that the LAN state, rather than the DMN, might play a central role in network interactions in AD. The probability that the DMN transitions to the LAN was higher in the AD relative to CH group. What's more, the VisN state, which in CH individuals showed the highest probability of transitioning to the DMN, in AD patients showed lower probability of transitioning to the DMN, but together with three other networks, showed higher probability of transitioning to the LAN.

Overall, our results suggest that synchrony disruptions in the DMN might have been a key contributor to the abnormalities observed in the prior MEG and EEG studies of AD. Capacity of spontaneous large-scale synchrony in this widely distributed network may be highly susceptible to the pathological changes in the aging brain. Because source modeling of the MEG signal involves inherent uncertainty (Dale and Sereno, 1993; Gross et al., 2013; Troebinger et al., 2014), it will be critical to obtain converging evidence for anatomic localizations of the transient neural abnormalities in AD in the future research. For instance, by recording EEG and fMRI simultaneously, it might be possible to directly show spatiotemporal correspondence between the slow electrophysiological/BOLD fluctuations and the occurrence of the fast electrophysiological HMM states. Additionally, developing methods for using the typical scalp topographies of the DMN and other HMM states as templates for parsing sensor-space EEG datasets may have promise in the clinical biomarker research. EEG can be conveniently and inexpensively acquired in the clinic, and with newly-optimized analysis methods, can yield sensitive indicators of neural network health in the clinical trial and other studies.

## 5 Conclusions

Through a novel application of mathematical modeling to the estimates of the electrophysiological activity in the brain during a resting MEG scan, we observed several types of abnormalities in the patterns of transient intrinsic synchronizations within large-scale neural networks in patients with AD. One of the most prominent functional brain networks, the DMN, which may play an important role in memory and other higher cognitive functions, was affected to the highest degree. Disrupted capacity to intrinsically synchronize cortical activity in the DMN, evident in the abnormally low state-specific oscillatory amplitudes and the reduced rate and stability of synchronizations, may underlie impairments in spontaneous memory retrieval and stimulus independent thought in patients with AD. Future research to determine the underlying physiological causes and consequences of this dysfunction at the cellular level may open new opportunities to discover improved treatments for this debilitating neurological condition.

## Figures and Tables

**Fig. A.1:**
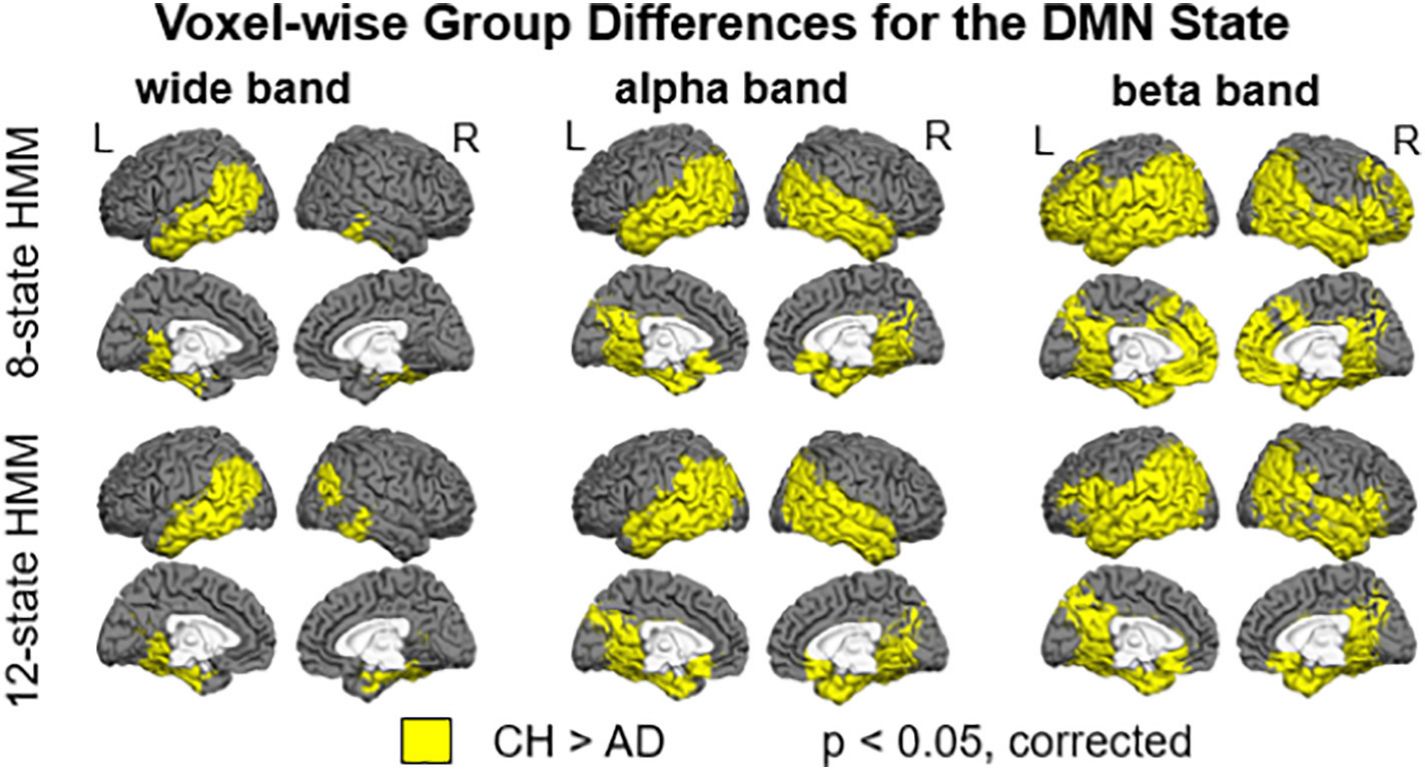
Maps of group differences in the voxel-wise DMN state-specific activity changes, obtained in the HMM inferred with 8 states (top) and 12 states (bottom). Brain voxels where the state-specific enhancement in the oscillatory amplitude was reduced in the AD relative to CH group are shown in yellow color for the wide band (4–30 Hz) in the left panel, the alpha band (8–12 Hz) in the central panel, and the beta band (15–30 Hz) in the right panel.

**Fig. A.2:**
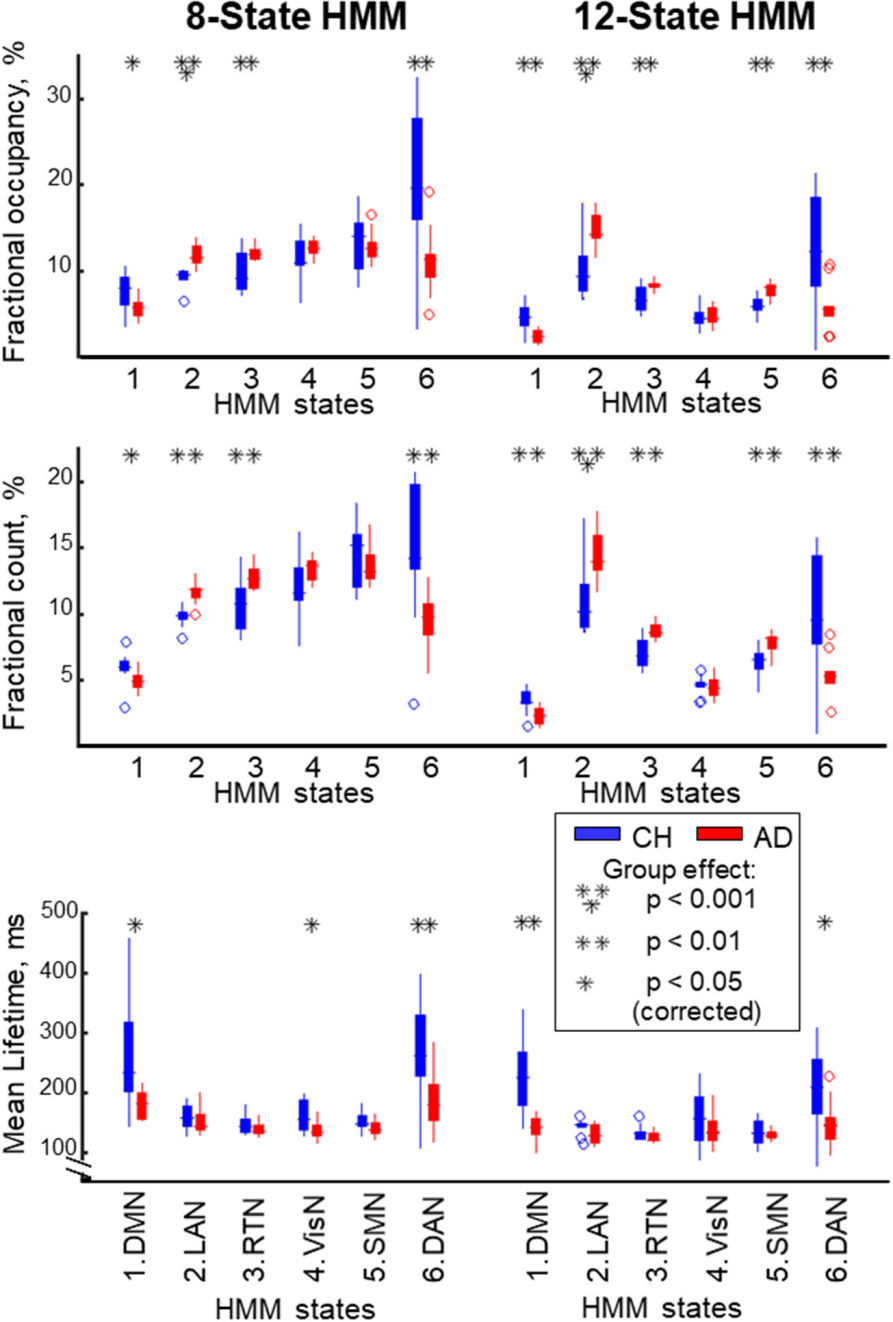
Group differences in temporal characteristics of the states inferred in the HMM with 8 states on the left and 12 states on the right. Boxplots illustrate value distributions (as described in Fig. 6) for fractional occupancy (top), fractional count (middle), and mean lifetime (bottom) for six salient states (labeled in the bottom), inferred in each model. Abbreviations are the same as in Fig. 2.

**Fig. B.1:**
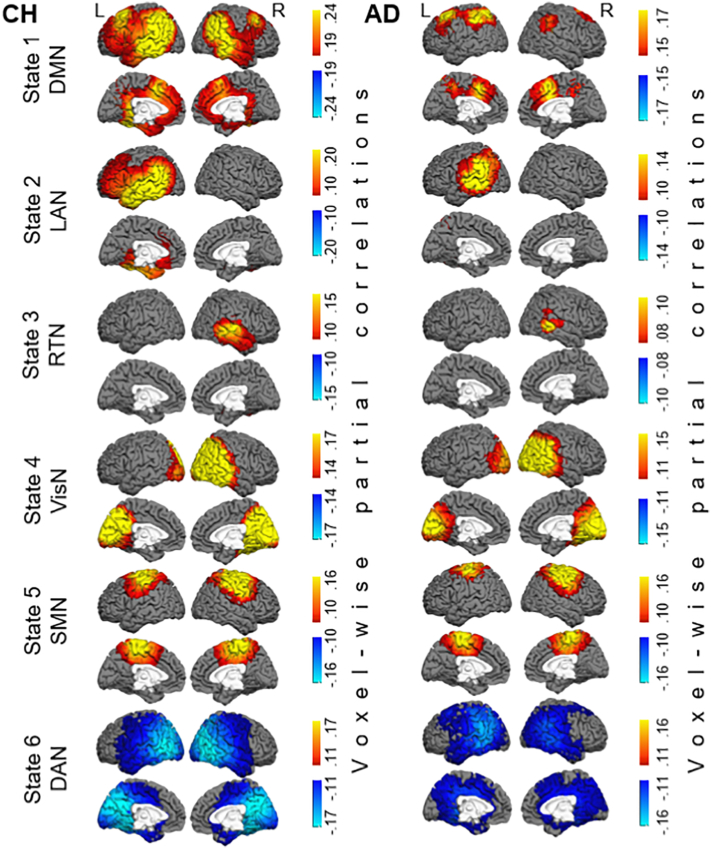
Maps of six salient states, which were inferred in the HMMs applied separately to the CH and AD group datasets. Maps of state-specific increases (in yellow/red/brown colors) and decreases (in blue color) in oscillatory amplitudes in the CH group are shown in the left panel and in the AD group in the right panel. Each map shows partial correlations between the state time-course and the oscillatory (4–30 Hz) amplitude envelopes in individual brain voxels. Abbreviations are the same as in Fig. 2.

**Fig. C.1:**
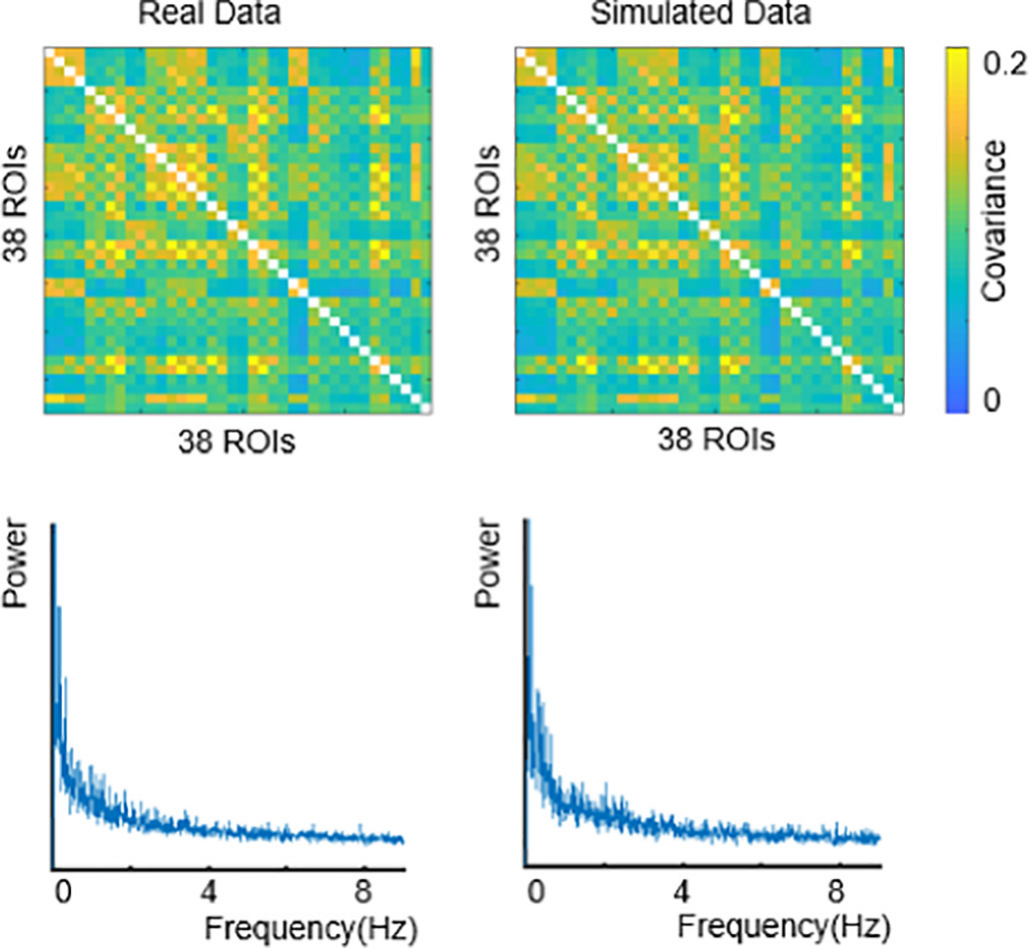
The full-length real and simulated multivariate datasets were matched in covariance and spectra. Shown are covariance matrices (top) and average power spectra (bottom) for the real CH dataset (left) and a corresponding simulated dataset (right).

**Fig. C.2:**
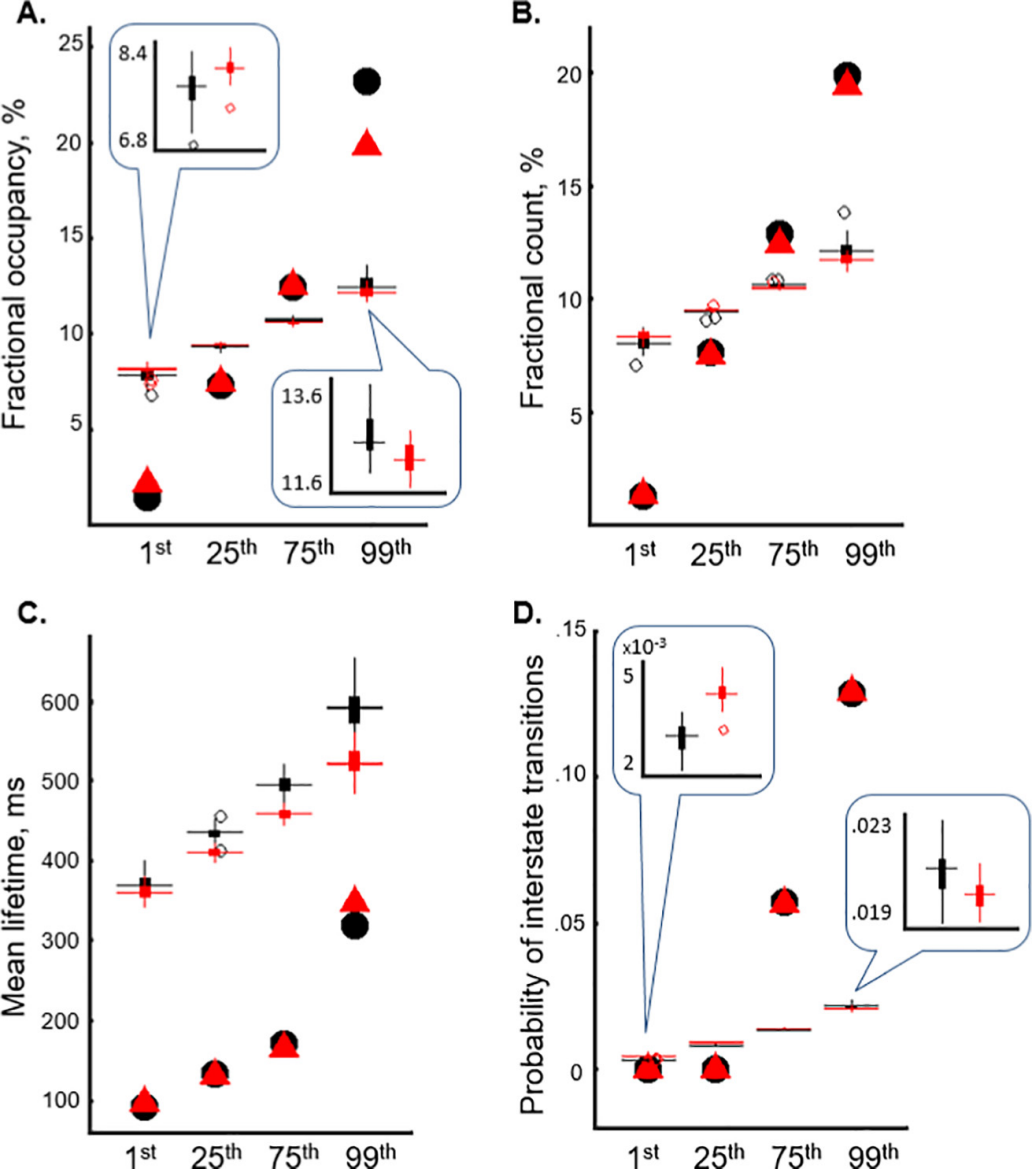
Temporal characteristics of 10 HMM states inferred in the simulated datasets compared to those in the real datasets. In each simulated multivariate dataset, fractional occupancy (A), fractional count (B), and mean lifetime (C) were computed for each state, for the sets of data points corresponding to the data of each CH participant. State-to-state transition probabilities (D) were also computed for the data-point sets corresponding to the data of each CH participant. We then determined values of these parameters corresponding to the 1st, 25th, 75th, and 99th percentiles in each simulated dataset. Boxplots show distributions of these values (as described in Fig. 6) across 50 datasets simulated based on the all-participant real dataset (in black) and 50 datasets simulated based on the CH-participant real dataset (in red). Corresponding parameter values in the all-participant real dataset (black solid circles) and in the CH-participant real dataset (red triangles) are shown for comparison. Inserts enlarge selective boxplots. The mean lifetimes of the HMM states were shorter in the real than simulated data (*p* < 0.05, corrected), and the within-participant variance in the other three temporal parameters between the HMM states was higher in the real than simulated data (*p* < 0.05, corrected).

**Fig. D.1:**
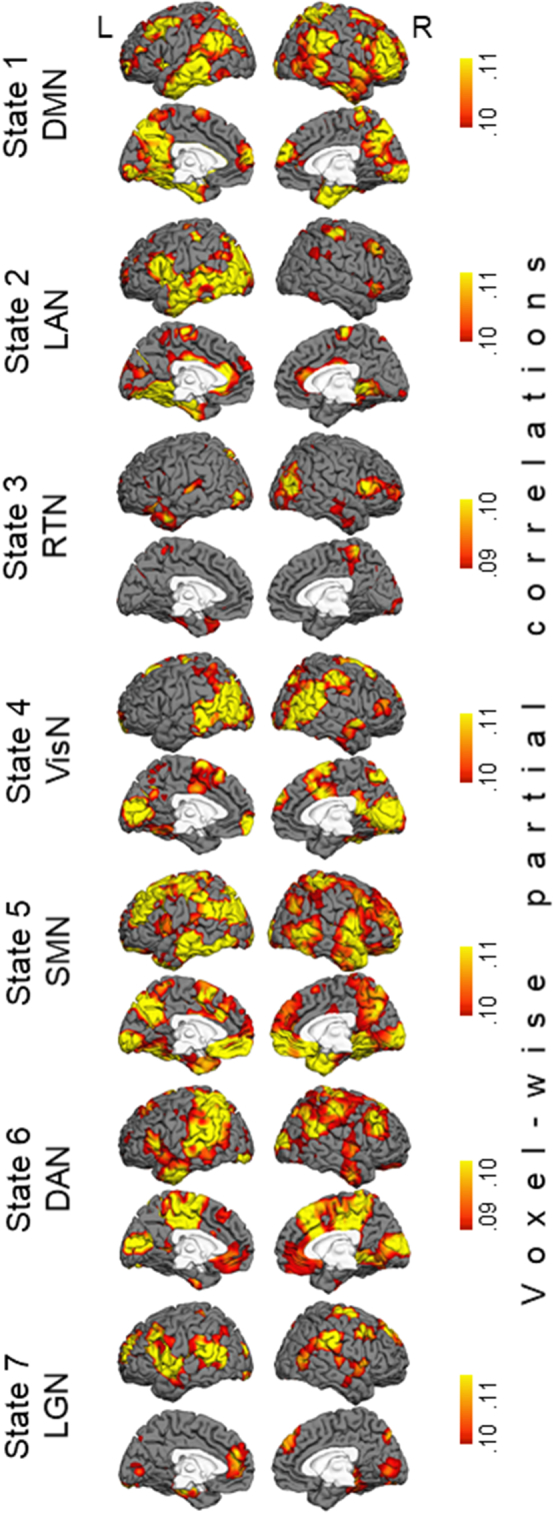
Voxel-wise maps of temporal coupling between the ultra-slow (<0.1 Hz) electrophysiological oscillations and the long timescale structure of seven salient HMM states in the CH group. Long timescale fluctuations in the rate of occurrence of each HMM state over time were quantified as changes in the proportion of time spent in the state within 5-sec-long (half-cycle length in 0.1 Hz oscillation) sliding windows. Each map shows the absolute values of partial correlations between the long timescale state time-course and the ultra-slow electrophysiological fluctuations in individual brain voxels. These effects were significantly lower in the control analysis with equalized state lifetimes (*p* < 0.05, corrected; note, Van de Ville et al., 2010 showed that equalizing state durations removed long-range dependencies in the electrophysiological data). Abbreviations are the same as in Fig. 2.

**Fig. E.1:**
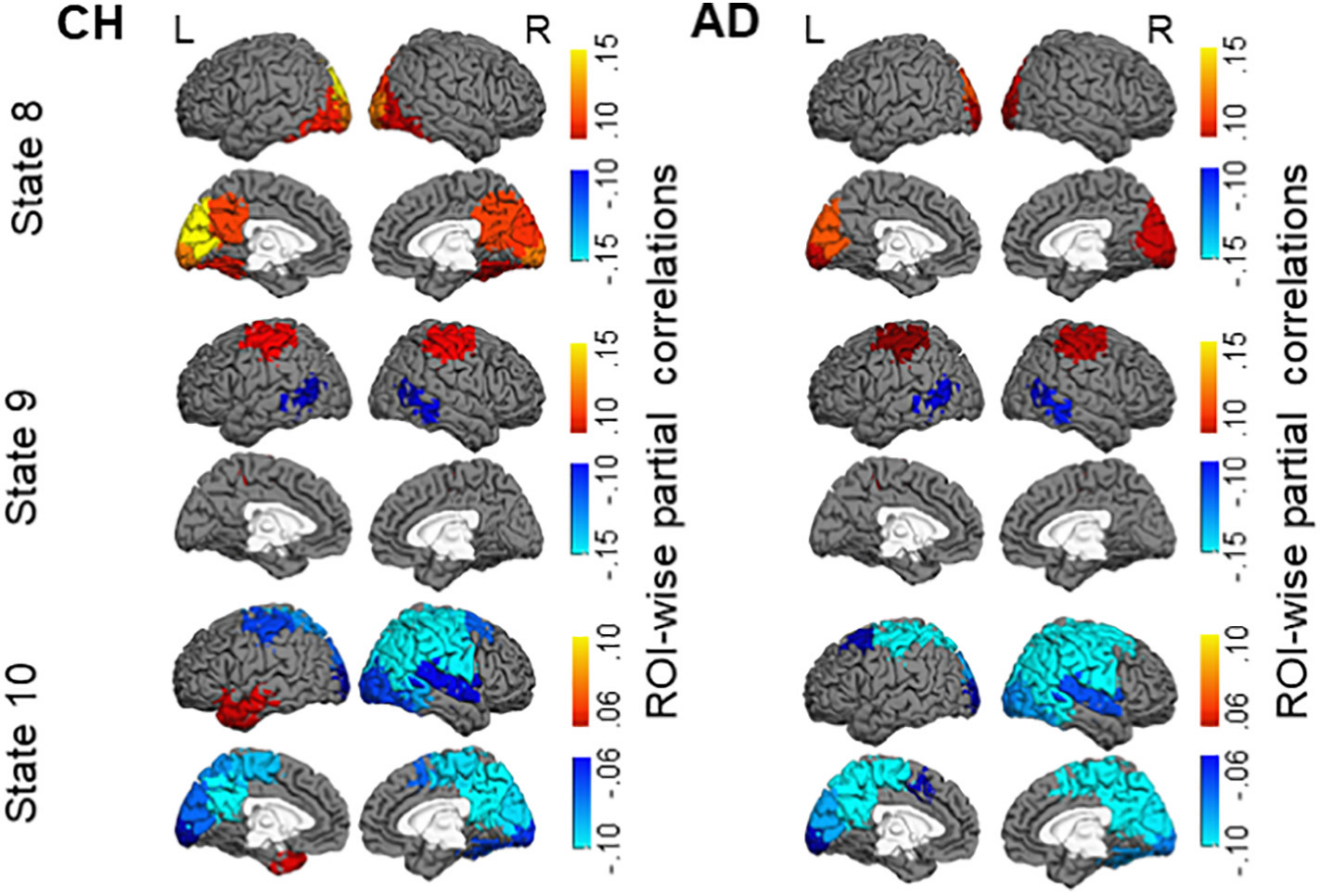
Maps of state-specific increases (in yellow/red/brown colors) and decreases (in blue color) in oscillatory amplitudes during three least salient (transitional) network states inferred by the HMM. Each map shows partial correlations between the state time-course and the oscillatory amplitude envelopes in the a priori ROIs. Maps in the CH group are shown in the left panel and in the AD group in the right panel. Abbreviations are the same as in Fig. 2.

## Appendix A HMM analyses with 8 and 12 states

We confirmed that the core results were not contingent on a specific number of states inferred by the HMM.

## Appendix B Group-specific HMM analyses with 10 states

We confirmed that the spatial topographies of the core states, observed in the combined HMM, could be replicated in the group-specific HMMs.

## Appendix C Real vs. stationary simulated data

Rich dynamics of the living brain could be described by diverse mathematical models. For instance, an alternative to an HMM with a relatively simple observation model of multiple states (Baker et al., 2014) may be a multivariate autoregressive model (MVAR) that can depict brain activity by a single state with a considerably more complex observation model. To evaluate the utility of the HMM in investigating the brain function, we compared the HMM between the real and surrogate data. In 100 simulations, we applied HMMs with 10 states to surrogate multivariate time-series characterized by stationary covariance and spectral properties that, across the entire time-series length, were matched to these of the real data. Because each of these simulated time-series exhibited activity that was largely as rich as the real data, it was expected that the HMMs with relatively simple state observation models would parse the data into distinct states. However, if the HMM states inferred in the real data reflect physiologically-driven, functional brain dynamics, it would be unlikely to detect comparable patterns in the surrogate datasets that were stationary by design.

A detailed description of the simulation algorithm can be found in (Laumann et al., 2017). Briefly, random normal deviates of the same dimensionality as the real dataset were multiplied in the spectral domain by the average power spectrum obtained from the full-length real dataset. These time-series were then projected onto the eigenvectors derived from the covariance matrix of the real multivariate time-courses. Thus, the resulting surrogate time-series were matched to the real data in the covariance structure and mean spectral content (cf. left vs. right panels in Fig. C.1), but being stationary by construction, could serve as a null against which to assess the nonstationary spatiotemporal architecture of the real dataset. In 50 of these simulations, the real data included the 38 ROI time-courses of the MEG-based activity estimates concatenated across all study participants. In the other 50 simulations, the real data were concatenated across the CH participants.

A sample MATLAB code for creating surrogate time-series can be downloaded from http://www.nil.wustl.edu/petersenschlaggar/Resources.html.

Comparisons of the HMM state inferences between the real and simulated data illustrate the value of this mathematical model in describing large-scale network behavior in the human brain. As is evident in Fig. C.2, the parameters of the HMM states inferred in each of the surrogate datasets lacked robust profiles characteristic of the real data.

## Appendix D Relevance of long timescale state sequence structure

The ultra-slow (~0.1 Hz) electrophysiological oscillations, detectable in the electrocorticography data in human participants as well as the local field potentials or neuronal calcium signals in the animal recordings (the data types with high spatiotemporal resolution), have been previously shown to exhibit both spatial and temporal correspondence to the spontaneous fMRI BOLD signal fluctuations (He et al., 2008; Matsui et al., 2016; Pan et al., 2013). Therefore, it was suggested that endogenous depolarizations of cortical apical dendrites, thought to underlie such ultra-slow potentials, might directly elicit changes in the BOLD signal (He et al., 2008). Additionally, the phase of the ultra-slow EEG potentials in human participants has been found to predict changes in the power of the higher-frequency (1–40 Hz) EEG oscillations, as well as the cognitive performance (e.g., detection of weak stimuli) (Monto et al., 2008). It is possible that by enhancing the cortical excitability, the dendritic depolarizations increase the probability of the transiently synchronized neuronal discharges, thus facilitating the processing of stimuli. Recent recordings of neuronal calcium signals in the mouse suggested how such interdependency between the ultra-slow and fast processes may be structured. The transient spatial patterns of synchronized activity might be embedded into the phase of global ultra-slow waves of spontaneous activity propagating across large-scale cortical networks (Matsui et al., 2016).

We hypothesized that the occurrence rates of the fast HMM states in human participants would be modulated by the intrinsic ultra-slow fluctuations in cortical excitability, which may be synchronized within the large-scale networks. In each network, the rate of coordinated fast changes in the oscillatory (4–30 Hz) amplitude would be expected to increase selectively during the negative shifts of the ultra-slow potentials, corresponding to the dendritic depolarizations. Fig. D.1 displays the maps of temporal concordance between the spontaneous ultra-slow electrophysiological fluctuations and the occurrence rates of HMM states in CH participants. The time-course for the occurrence rate fluctuations for each HMM state was quantified as changes in the proportion of time spent in the state within 5-s-long sliding windows (half a cycle length in 0.1 Hz oscillation). Shown are partial correlations (absolute values) between the ultra-slow potentials in each cortical voxel and the occurrence rates of each of the seven salient HMM states, inferred in the primary 10-state HMM (these coefficients were significantly higher than those in the control analysis with equalized state lifetimes, cf. Van de Ville et al., 2010). The spatial patterns of the ultra-slow/fast coupling in each map overlap the nodes of the DMN or other large-scale neural networks, and for the large part, are similar to the corresponding maps of the state-specific oscillatory (4–30 Hz) amplitude modulations (cf. Fig. 3). This selective spatial pattern has emerged even though the long-scale time-courses of the occurrence rates were temporally correlated between the HMM states (Fig. D.2), indicating that there was a slight but consistent shift in the phase of long timescale fluctuations between the large-scale neural networks. Only in the case of the highest positive correlation between the SMN and DMN time-courses, the spatial selectivity is less evident. The spatiotemporal coupling between the intrinsic ultra-slow potentials and the long timescale structure of the fast HMM states is in agreement with the possibility that dendritic depolarizations are a common underlying factor linking the HMM states and the spontaneous fMRI BOLD coactiviations.

There is also evidence that the fast timescale variance in the neural activity may modulate the BOLD response. Simultaneous fMRI and neuronal calcium imaging in the mouse have demonstrated that transient synchronizations in the functional networks (phase-locked to the ultra-slow activity) evoke spatially similar BOLD activity patterns and are necessary to sustain such spatial BOLD structure (Matsui et al., 2016). Furthermore, the neural processes in different high-frequency bands (e.g., alpha, beta, gamma), captured in the local field potentials in the monkey, have been found to correlate with distinct properties of the simultaneously acquired BOLD signal (Magri et al., 2012). Because fast changes in neural synchrony were shown to exhibit self-similar dynamics spanning the scales from a few milliseconds to several seconds, it has been argued that equivalent neural processes may be observed through different temporal filters by the fast electrophysiological and the slow fMRI recordings (Bellay et al., 2015; Hahn et al., 2017; Van de Ville et al., 2010). This scale-free property could explain the spatial correspondence between the short timescale abnormalities found in AD in the present investigation and the slower effects in the prior fMRI studies, both lines of research localizing the functional deficits in AD to the DMN. We confirmed that the transient activity abnormalities in AD in the regions overlapping the DMN were unlikely a mere consequence of the disrupted long timescale scheduling, related to the ultra-slow parameters of cortical excitability. Fig. D.3 illustrates, for each state, the patterns of short-scale abnormalities detected selectively during the time-intervals exhibiting an increased state occurrence rate (identified based on the slow time-courses of the occurrence rate fluctuations: values >50th percentile). During such time-windows of presumed high local cortical excitability, the DMN state in AD, relative to that in CH, showed bilateral reductions in the 4–30 Hz neural oscillations in the temporal and parietal cortices, as well as disruptions in the naturally irregular temporal patterns of visits to the state.

Application of the HMM to the source-localized MEG data infers short-range regularities, but also does not preclude modeling the state time-courses containing data-driven long-range dependencies. It is possible that such long timescale scheduling of the transient network states yields patterns corresponding to network synchronizations measurable by fMRI. Whether the fast processes that we found abnormal in AD have a corresponding signature in the fMRI modality is a question that could be addressed in future research, for instance, by directly linking between the electrophysiological and fMRI modalities via series of simultaneous EEG/fMRI and EEG/MEG recordings.
